# Supplementary material for: Process-evaluation of forest aerosol-cloud-climate feedback shows clear evidence from observations and large uncertainty in models
Source: Nat Commun. 2024 Feb 7;15:969. doi: 10.1038/s41467-024-45001-y (PMC10850362; doi:10.1038/s41467-024-45001-y)
Supplement: Supplementary file 1 — Supplementary Information [file 41467_2024_45001_MOESM1_ESM.pdf]

Supplementary for: Aerosol-cloud-climate feedbacks over forests: Clear evidence from observations, large uncertainty in Earth System Models

Sara M. Blichner[1,2,*], Taina Yli-Juuti[3], Tero Mielonen[4], Christopher Pöhlker[5], Eemeli Holopainen[4,§], Liine Heikkinen[1,2], Claudia Mohr[1,2,‡], Paulo Artaxo[6], Samara Carbone[7], Bruno Backes Meller[6], Cléo Quaresma Dias-Júnior[8], Markku Kulmala[9], Tuukka Petäjä[9], Catherine E. Scott[10], Carl Svenhag[11], Lars Nieradzik[12], Moa Sporre[11], Daniel Partridge[13], Emanuele Tovazzi[13], Annele Virtanen[3], Harri Kokkola[3,4], and Ilona Riipinen[1,2]

[1]Stockholm University, Department of Environmental Science, Stockholm, SE-106 91, Sweden
[2]Stockholm University, Bolin Centre for Climate Research, Stockholm, Sweden
[3]University of Eastern Finland, Department of Technical Physics, 70211 Kuopio, Finland
[4]Finnish Meteorological Institute, Kuopio, FI-70211, Finland
[5]Max Planck Institute for Chemistry, Multiphase Chemistry Dept., 55128 Mainz, Germany
[6]Universidade de Sao Paulo, Instituto de Fisica, 05508–090 Sao Paulo, Brazil
[7]Federal University of Uberlândia, Institute of Agrarian Sciences, Uberlândia, MG, Brazil
[8]Federal Institute of Pará, Department of Physics, Belém, Pará, Brazil
[9]University of Helsinki, Institute for Atmospheric and Earth System Research (INAR), Helsinki, Finland
[10]University of Leeds, School of Earth and Environment, Leeds, LS2 9JT, UK
[11]Lund University, Department of Physics, 221–00 Lund, Sweden
[12]Lund University, Dept of Physical Geography and Ecosystem Science, 221–00 Lund, Sweden
[13]University of Exeter, Department of Mathematics and Statistics, Exeter, United Kingdom
[*]sara.blichner@aces.su.se
[‡]Now at: Department of Environmental System Science, ETH Zurich and Laboratory of Atmospheric Chemistry, Paul Scherrer Institute, Villigen, Switzerland
[§]Now at: Institute for Chemical Engineering Sciences, Foundation for Research and Technology - Hellas (FORTH/ICE-HT), Patras, Greece.

▪

Contents

S1 Sensitivity to choice of season for ATTO 3

S2 Sensitivity to choice of level at ATTO 7

S3 Total feedback strength for COT 8

S4 Modelled emissions 9

S4.1 SMEAR-II 9

S4.2 ATTO 11

S5 Regressions 14

S5.1 Regression coefficients for temperature to OA and OA to N_x 14

S6 Regression with linear lines for OA to N_x 16

S6.1 Residuals for fitted regressions 17

Temperature to OA • OA mass to N_x

S7 Cloud properties 24

S7.1 Overview cloud properties through the year at ATTO 24

S7.2 Cloud impacts of high versus low CCN (N_{50} and N_{100}) 25

S7.3 Distributions of cloud properties 27

S8 Detailed discussion on regression coefficients 28

S8.1 Temperature to organic aerosol mass 28

Yield tuning: An example with NorESM

S9 Basic aerosol evaluation: SMEAR-II 30

S9.1 Organic aerosol mass 30

S9.2 Aerosol concentrations: N50, N100 and N200 32

S9.3 Time series N50, N100, N200 36

S10 Basic aerosol evaluation: ATTO 39

S10.1 Organic aerosol mass 39

S10.2 Aerosol concentrations: N50, N100 and N200 43

S10.3 ATTO: Time series N50, N100, N200 46

S11 Model descriptions 48

S11.1 The Norwegian Earth System model 48

S11.2 EC-Earth 50

S11.3 ECHAM-SALSA 50

S11.4 UKESM 50

S12 Model data post-processing 51

EC-Earth • UKESM • NorESM • ECHAM-SALSA

S13 Supplementary references 52

S1 Sensitivity to choice of season for ATTO

For SMEAR-II, we based our analysis on [1] and the choice of season was therefore given. For ATTO, on the other hand, we choose the season based on (1) when biogenic SOA is expected to dominate the OA (wet season) [2] and (2) when there is not strong seasonal changes in cloud properties (see Fig. S29). However, we find that our results do not change in character depending on which months are chosen, as can be seen in Fig. S1–S3 which show results from similar analysis as presented in Fig. 2–4 but for different choice of months.

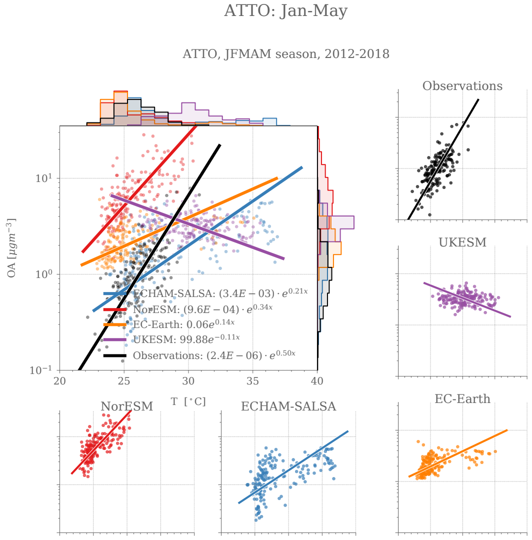

(a) Jan–May

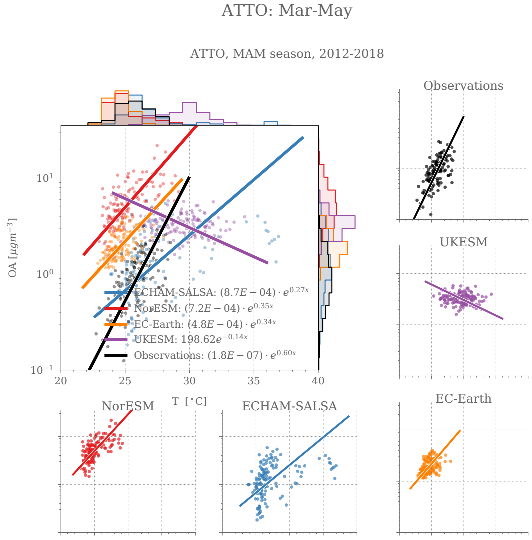

(b) Mar–May

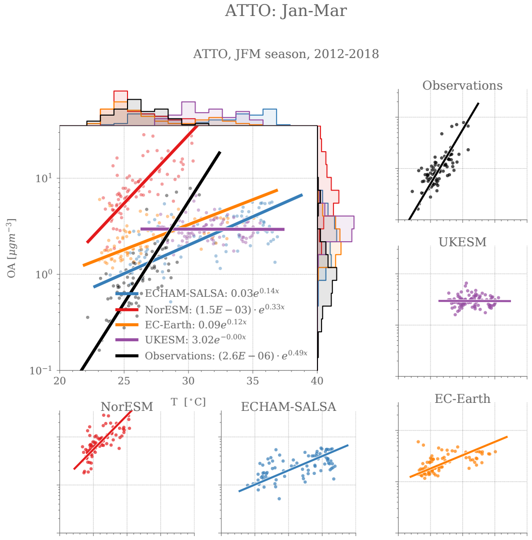

(c) Jan–Mar

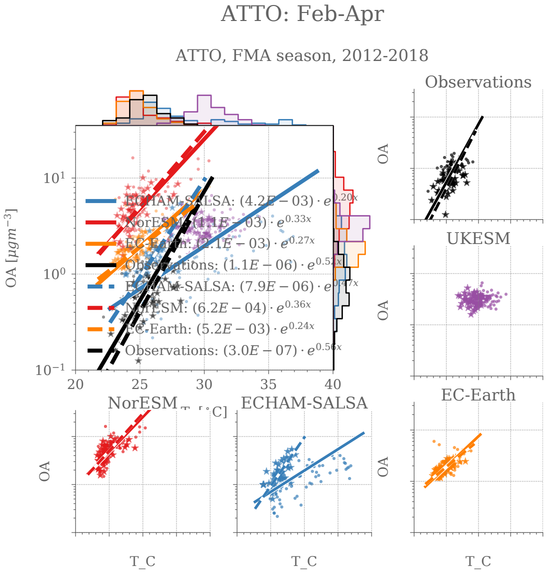

(d) Feb–Apr

Figure S1. ATTO: Same as Fig. 2, but only for ATTO and varying the months. Relationship between temperature and OA mass concentration in ESMs (blue, red, orange and purple for ECHAM-SALSA, NorESM, EC-Earth and UKESM respectively) and observations (black). The main plot for each choice of months shows all the daily median values of temperature and OA mass and the least-square regression to an exponential function for the observations and nudged model predictions for the same periods (see Methods for details). Histograms of the observed and predicted values are shown on the top and right sides of the main plots. The smaller plots display the same information as the large plots but for each data source separately. The ESM grid box covering the station (nearest-neighbour) is chosen for evaluation. Months included in each sub-figure are a) January-May, b) March-May, c) January-March and d) February-April.

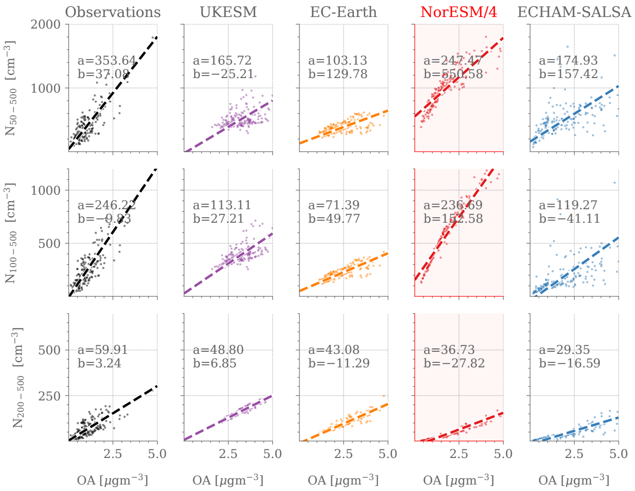

(a) Jan–May

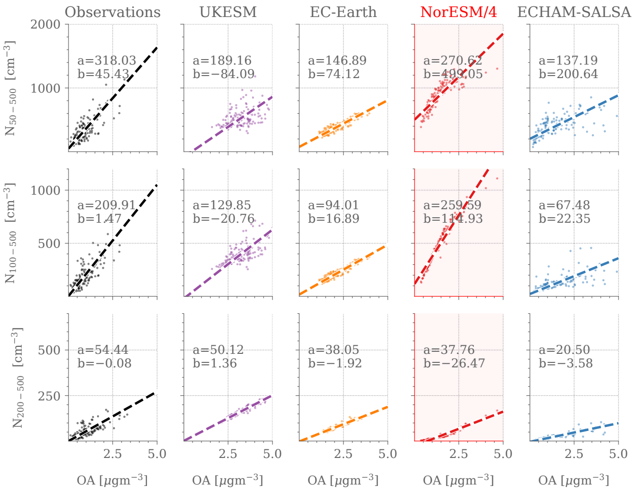

(b) Mar–May

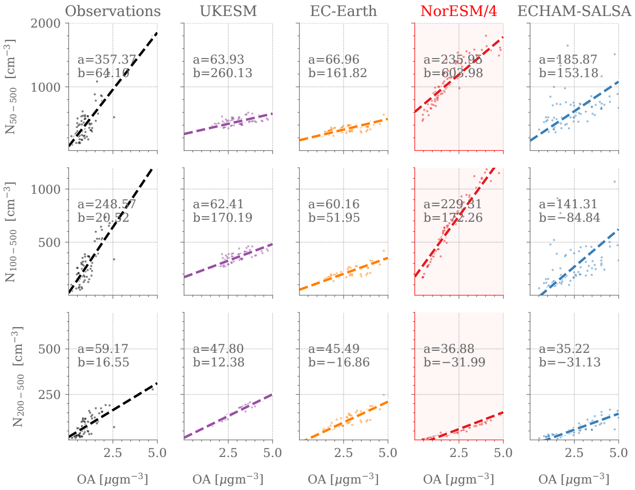

(c) Jan–Mar

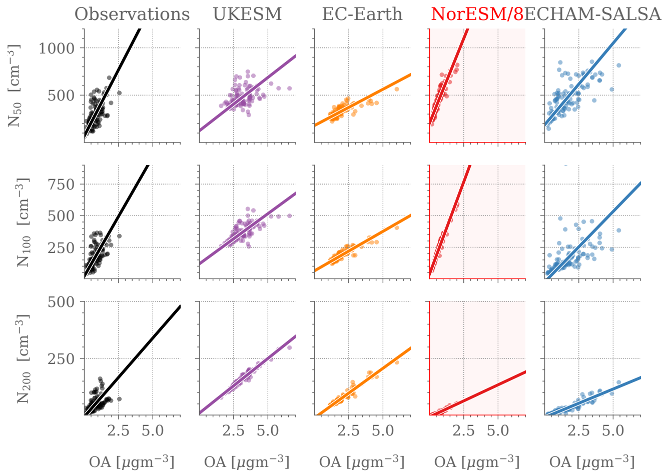

(d) Feb–Apr

Figure S2. Same as Fig. 3, but for different choices of months and only for ATTO. The relationship between daily median OA mass concentration and the number concentration of particles larger than 50 nm (N_{50}), 100 nm (N_{100}) and 200 nm (N_{200}). The lines show the orthogonal distance regression to a linear function ($ax+b$) (see Methods for details). Note that the NorESM values are divided by four both for OA mass and the particle number concentration (marked by the red axis and background colour of the plot) for figures a-c and divided by eight for subplot d). Months included in each sub-figure are a) January-May, b) March-May, c) January-March and d) February-April.

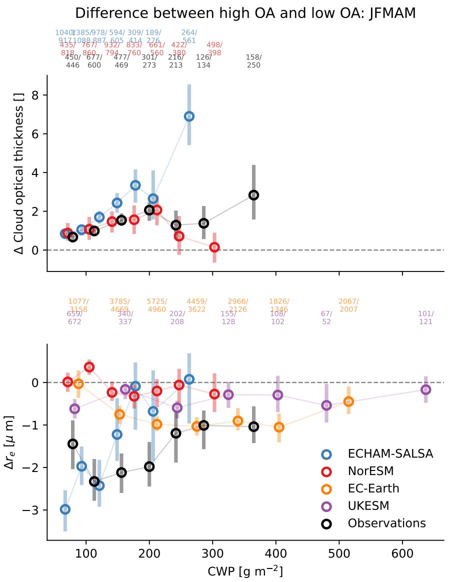

(a) Jan–May

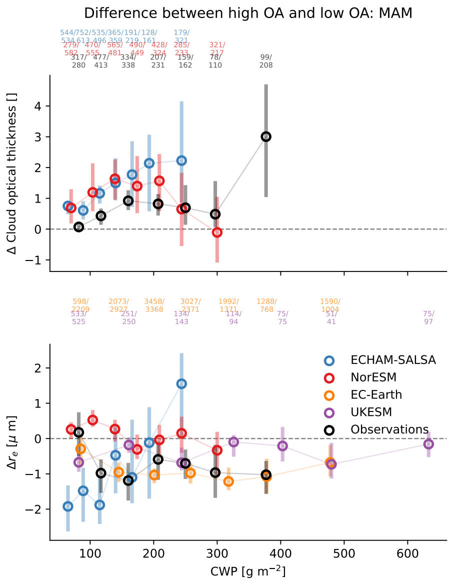

(b) Mar–May

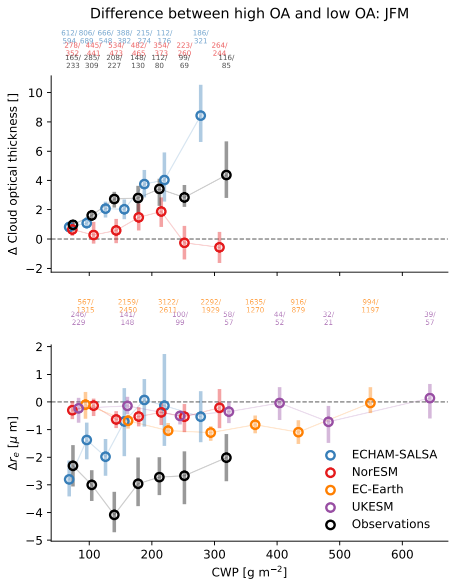

(c) Jan–Mar

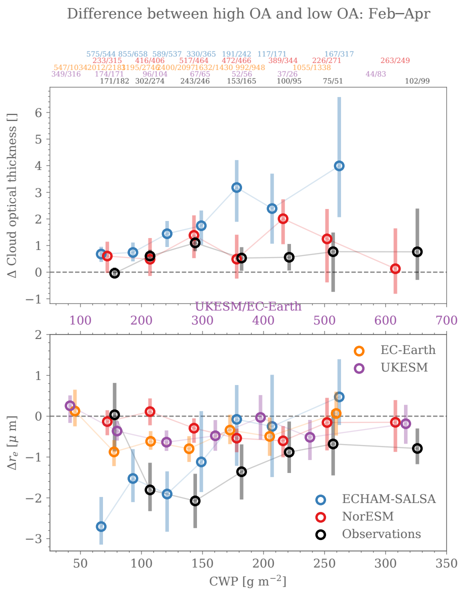

(d) Feb–Apr

Figure S3. ATTO: Same as Fig. 4, but for different months and only for ATTO. The difference in median cloud optical thickness (top) and cloud droplet effective radius (bottom) between high OA (above 67th percentile) days and low OA (below 34th percentile) days. The uncertainties marked by the bars and are the 2.5th to 97.5th percentile of the median calculated by bootstrapping and the percentile method (see Methods section). The numbers at the top signify the number of data points in low/high OA in each CWP bin. Note that for UKESM and EC-Earth, COT was not available as output. Months included in each sub-figure are a) January-May, b) March-May and c) January-March.

S2 Sensitivity to choice of level at ATTO

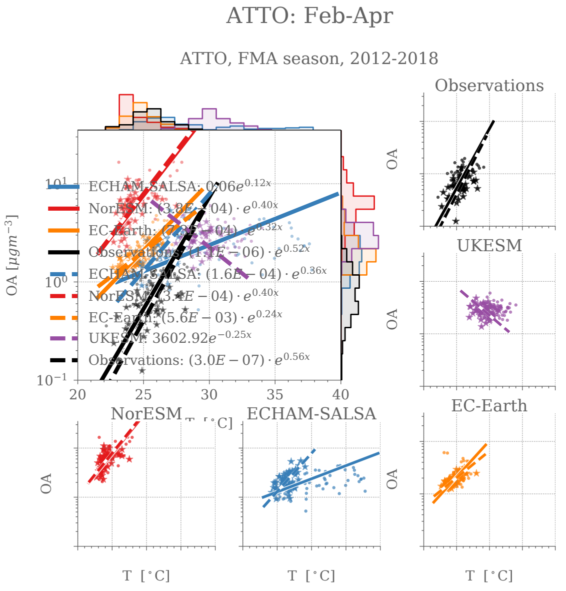

Figure S4. Same as Fig. 2, but with using the level closest to the surface, instead of the second lowest. Relationship between temperature and organic aerosol (OA) mass concentration in Earth System Models (ESMs) (blue, red, orange and purple for ECHAM-SALSA, NorESM, EC-Earth and UKESM, respectively) and observations (black) at SMEAR-II (left panel) and ATTO (right panel) during periods where biogenic SOA is known to dominate the OA budget (February–April at ATTO). The main plot shows all the daily median values of temperature and OA mass and the least-square regression to an exponential function for the observations and nudged model predictions for the same periods (see Methods for details). Histograms of the observed and predicted values are shown on the top (for temperature)and right (for OA) side of the main plots. The smaller plots display the same information as the large plot but for each data source separately. The ESM grid box covering the station (nearest-neighbour) is chosen for evaluation. For ATTO, star symbols and dashed lines indicate values when 2015/2016 are excluded from the data.

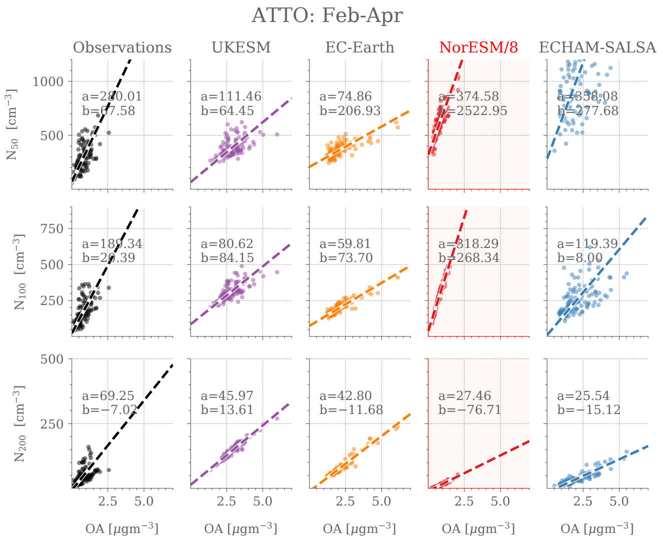

Figure S5. Same as Fig. 3, but with using the level closest to the surface, instead of the second lowest. The relationship between daily median organic aerosol (OA) mass concentration and the number concentration of particles larger than 50 nm (N_{50}), 100 nm (N_{100}) and 200 nm (N_{200}) for ATTO in February–April (right). The size distribution measurements only go up to 500 nm, so we use the same intervals for the models. The lines show the least-square regression to a logarithmic function $(a+b\ln(c+x))$ for N_{50} and N_{100} at SMEAR-II and the orthogonal distance regression to a linear function for all the others $(ax+b)$ (see Methods for details). NB: The axis limits for NorESM are eight times those of EC-Earth, ECHAM-SALSA and UKESM (indicated by the red/purple axis and background color of the plot)

S3 Total feedback strength for COT

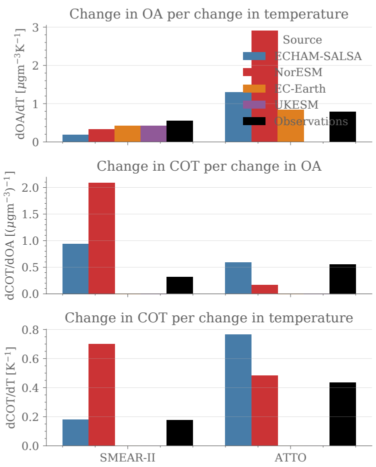

Figure S6. Estimated strength of the terms in the feedback loop for the change in OA mass per temperature (top panel), the change in COT per change in OA mass (middle panel), and finally the product of these two terms, $\frac{dCOT}{dT} = \frac{dOA}{dT} \cdot \frac{dCOT}{dOA}$ (bottom panel). The change in OA mass per change in temperature is estimated in accordance with the exponential fit for each model and with a 3-degree temperature perturbation from the current median (in accordance with the best estimate for climate sensitivity, [3]). The change in COT from change in OA mass is estimated by doing a weighted average over the bins in Fig. 4.

S4 Modelled emissions

S4.1 SMEAR-II

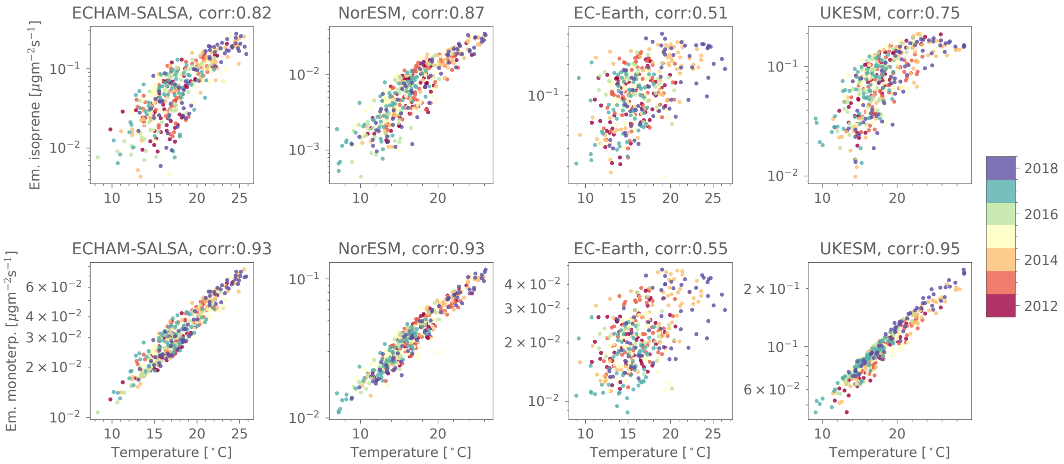

Figure S7. SMEAR-II Jul & Aug: The relationship between temperature (x-axis) and BVOC emissions in the four models: Isoprene (y-axis, top) and monoterpene (y-axis, bottom). The title gives the model and the Pearson correlation coefficient for each plot. These plots include all data, i.e. they are not filtered by when the observations are available. Each dot represents a daily median value.

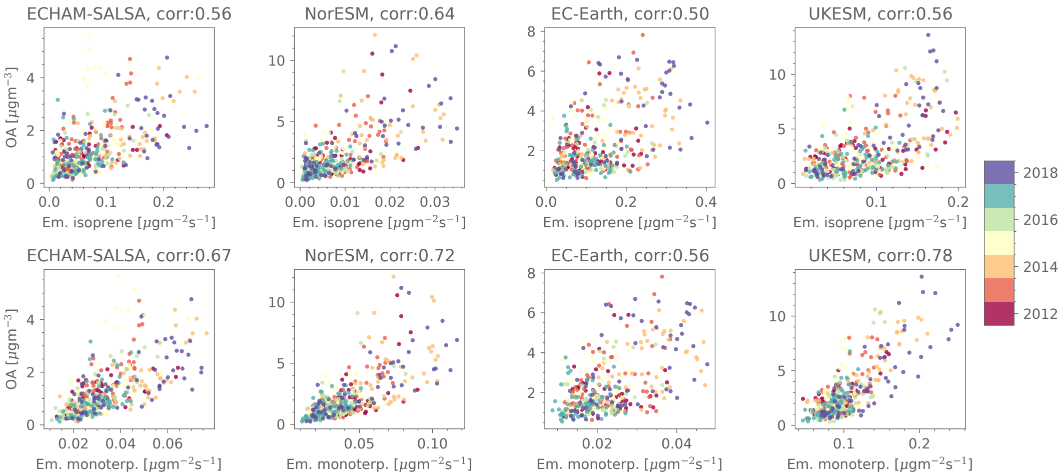

Figure S8. SMEAR-II Jul & Aug: The relationship between emissions of isoprene (x-axis, top) and monoterpene (x-axis, bottom) and OA mass (y-axis) in the four models. The title gives the model and the Pearson correlation coefficient for each plot. These plots include all data, i.e. they are not filtered by when the observations are available. Each dot represents a daily median value. Note that in UKESM, only monoterpene contributes to SOA formation.

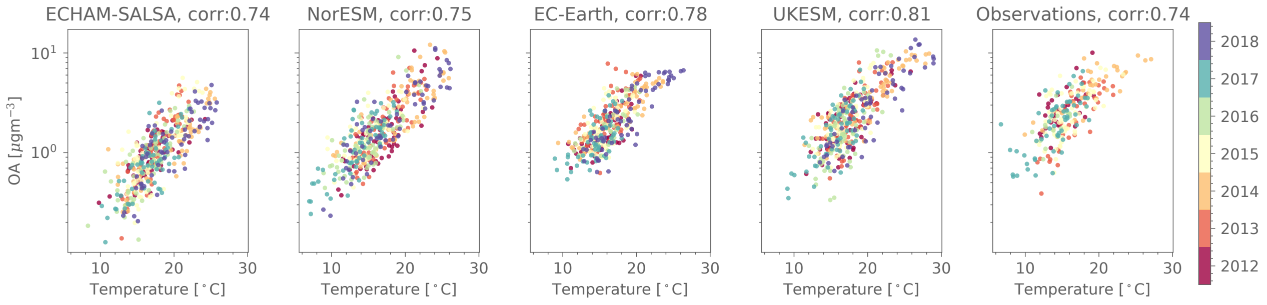

Figure S9. SMEAR-II July & August: The relationship between temperature (x-axis') and OA mass (y-axis) in the four models and the observations. The colour of the dots signifies the year. The title gives the model and the Pearson correlation coefficient for each plot. These plots include all data, i.e. they are not filtered by when the observations are available. Each dot represents a daily median value.

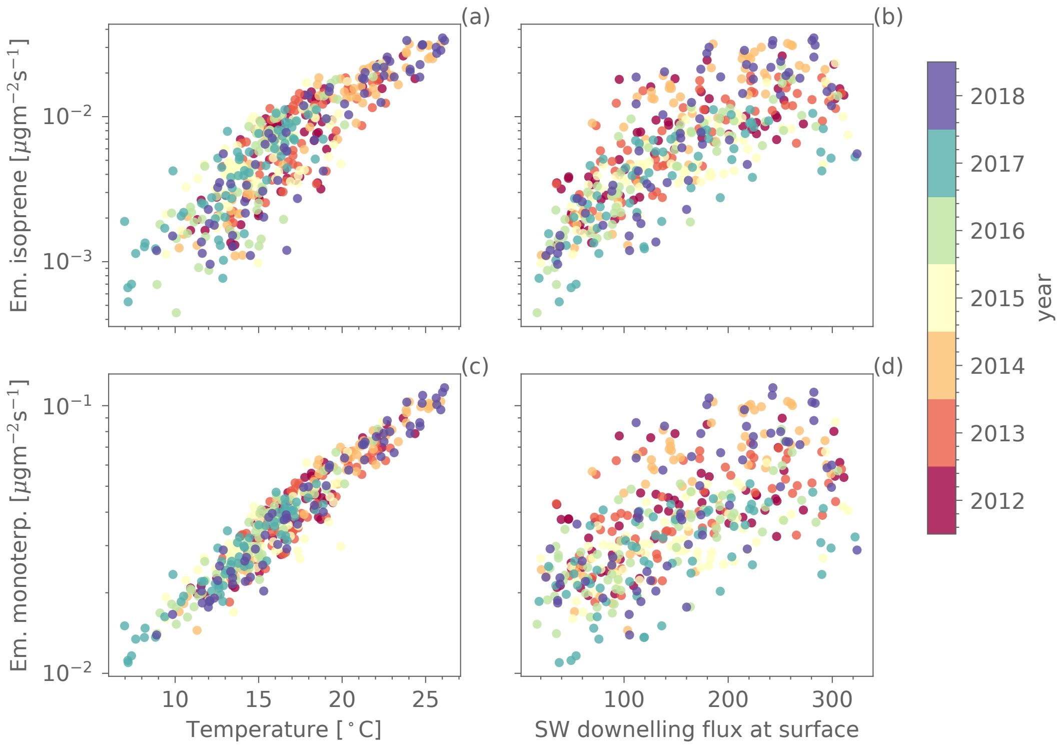

Figure S10. SMEAR-II in July & Aug: The relationship between temperature (x-axis, left) and incoming short wave (SW) radiation at the surface (x-axis, right) and BVOC emissions in NorESM: Isoprene (y-axis, top) and monoterpene (y-axis, bottom). These plots include all data, i.e. they are not filtered by when the observations are available. Each dot represents a daily median value.

Figure S7 shows the relationship between temperature and BVOC emissions divided into isoprene and monoterpene at SMEAR-II during July and August. These show a very clear, and very similar, relationship for all the models using MEGAN2.1 (UKESM and NorESM), and a slightly less clear one for EC-Earth (using emissions from LPJ-Guess). The slope is, though, lower for UKESM than the other models. Figure S8 shows the relationship between BVOC emissions (isoprene and monoterpene) and the concentration of OA mass in the models. These results show clear relationships, though for isoprene the relationship may be mainly driven by similar emission drivers as monoterpene as SOA production from isoprene is quite weak in the boreal forest (see yields in section S11). Also note that in UKESM, isoprene does not contribute to SOA formation at all. Finally, the relationships between temperature and OA mass (as in Fig. 2) are repeated in Figure S9 but this time coloured by year to show that there does not seem to be any large perturbations based on the year (which is different for ATTO – see next section). Finally, to investigate the impact of radiation versus temperature on emissions, we show in Fig. S10 the relationship between

temperature and short wave radiation at the surface and BVOC emissions. This is only shown for NorESM because we did not have the output to do these plots for the other models. It reveals that both monoterpene and isoprene emissions are more tightly linked to temperatures than radiation in NorESM for this boreal forest environment during summer.

S4.2 ATTO

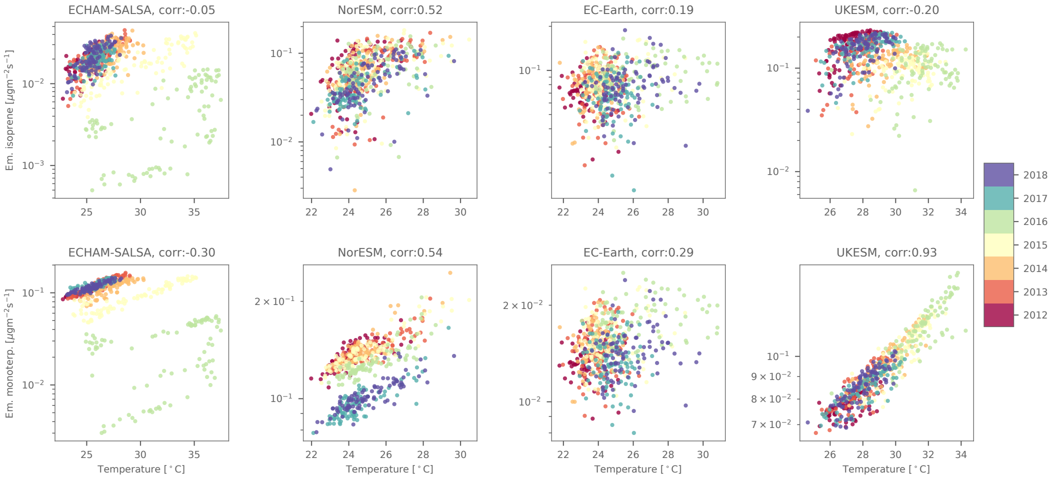

Figure S11. ATTO in Feb–Apr: The relationship between temperature (x-axis) and isoprene (y-axis, top) and monoterpene (y-axis, bottom) for the four models. The title gives the model and the Pearson correlation coefficient for each plot. These plots include all data, i.e. they are not filtered by when the observations are available. Each dot represents a daily median value.

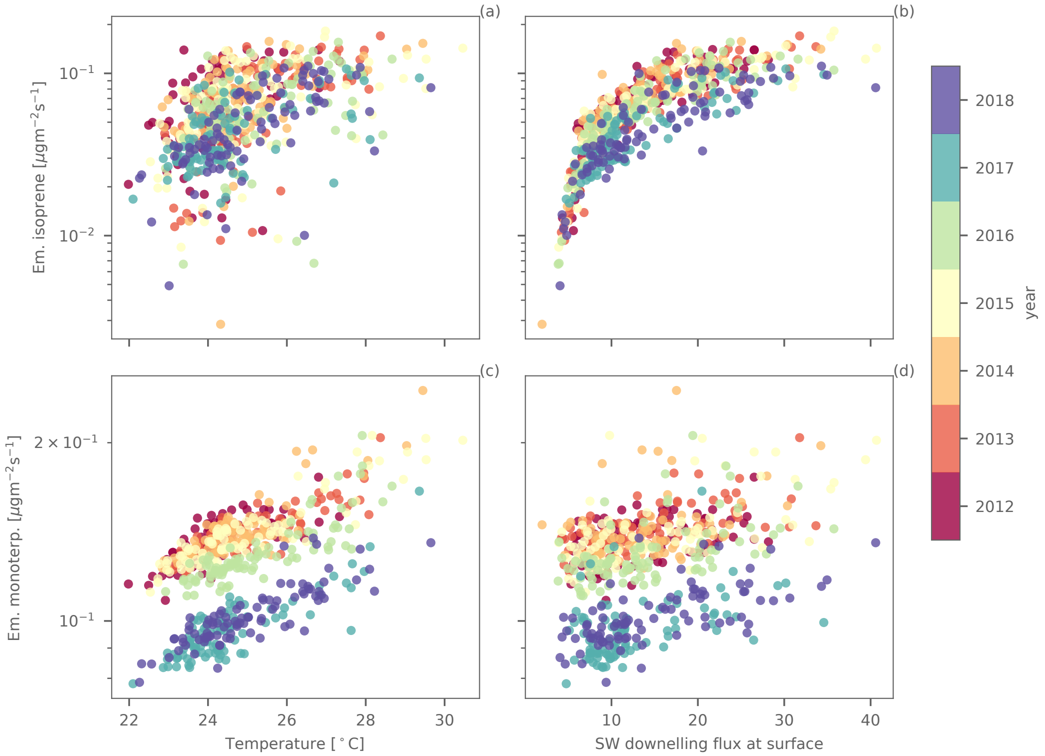

Figure S12. ATTO in Feb–Apr, NorESM: The relationship between temperature (x-axis, left) and incoming short wave radiation at the surface (x-axis, right) and isoprene (y-axis, top) and monoterpene (y-axis, bottom). These plots include all data, i.e. they are not filtered by when the observations are available. Each dot represents a daily median value.

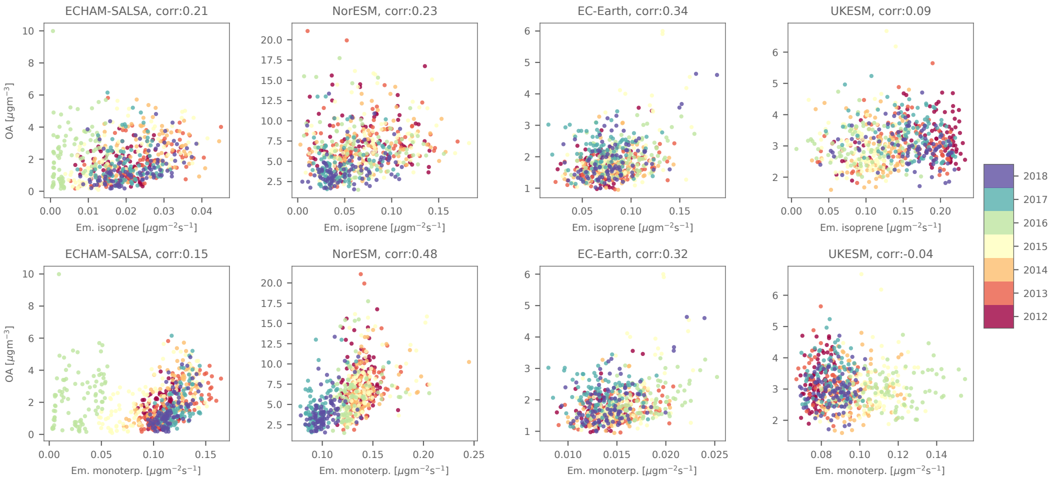

Figure S13. ATTO February – April: Relationship between emissions of isoprene and monoterpene (x-axis') and OA mass (y-axis) for the four models. The colour of the dots signifies the year. The title gives the model and the Pearson correlation coefficient for each plot. These plots include all data, i.e. they are not filtered by when the observations are available. Each dot represents a daily median value. Note that in UKESM, only monoterpene contributes to SOA formation.

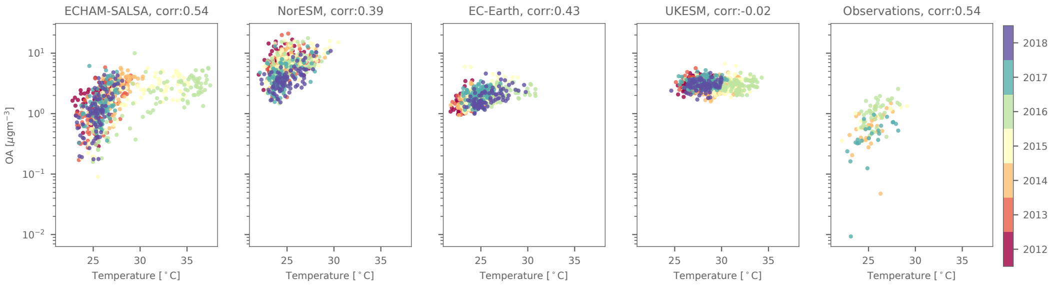

Figure S14. ATTO February – April: Relationship between temperature (x-axis') and OA mass (y-axis) for the four models and the observations. The colour of the dots signifies the year. The title gives the model and the Pearson correlation coefficient for each plot. These plots include all data, i.e. they are not filtered by when the observations are available. Each dot represents a daily median value. Similar to Fig. 2, but including all model data and has colouring by year.

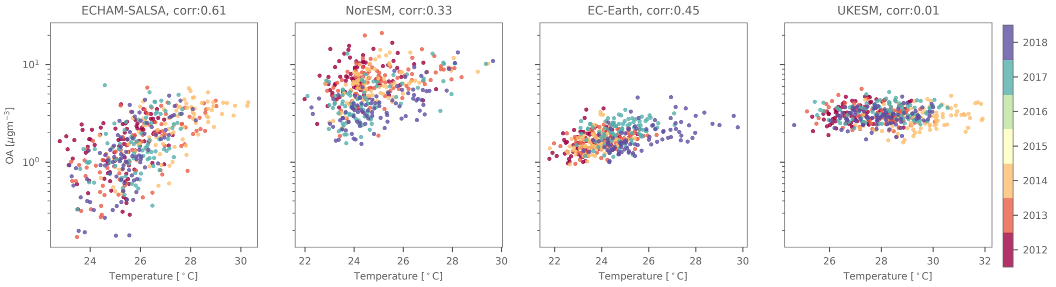

Figure S15. ATTO February – April excluding years 2015 and 2016: Relationship between temperature (x-axis') and OA mass (y-axis) for the four models. The colour of the dots signifies the year. The title gives the model and the Pearson correlation coefficient for each plot. These plots include all data, i.e. they are not filtered by when the observations are available. Each dot represents a daily median value.

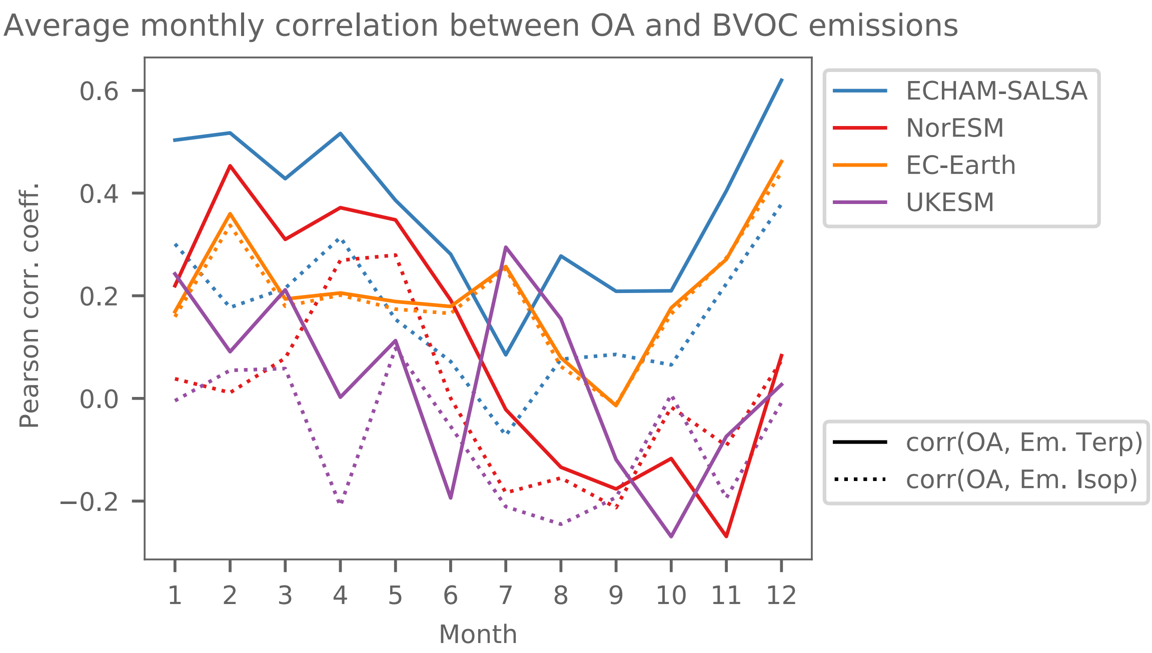

Figure S16. ATTO: Pearson correlation coefficient between emissions of BVOCs (isoprene and monoterpene) and OA mass per month for the four models. The correlation is first calculated in each month and then averaged. The solid lines are OA and monoterpene emissions and the dotted lines are OA and isoprene emission. These plots include all data, i.e. they are not filtered by when the observations are available. The input data is daily medians.

Figure S11 shows the relationship between temperature and BVOC emissions divided into isoprene and monoterpene at ATTO during Feb–Apr. The colour of the dot signifies the year and reveals that there is significant difference in how the models treat interannual variability. ECHAM-SALSA and NorESM stand out here with a particularly strong interannual variability. For both the relationship between temperature and emissions is much clearer within a single year, but is somewhat distorted by what appears to be another influence on the interannual level. For NorESM, we could confirm that this is due to a significant reduction in biomass due to forest fires when going from the historical simulation configuration (up to year 2014) to the SSP-2-4.5 configuration (not shown here). The reduction in LAI thus reduces the baseline emission upon which the temperature impact comes. Something similar might be happening for ECHAM-SALSA – there are reduced emissions in 2015 and 2016 – but in this case the emissions seem to recover fast (by 2018 they are similar to before). Note however, that the high OA mass values for 2015 and 2016 are at extremely high temperatures (see also Fig. S11) and if these were excluded, the BVOC emission to OA mass relationship would appear less noisy. EC-Earth has a weaker dependency on temperature for both emitted species compared to the other three models. Finally, UKESM has a very strong relationship between temperature and monoterpene emissions and no large impact of the year beyond what is expected from the temperature, though a very weak one, or even a negative one, for isoprene emissions.

We show in Fig. S12 the relationship between temperature and short wave radiation at the surface and BVOC emissions, again only for NorESM due to data availability constraints. This reveals that radiation has a stronger effect, in particular on isoprene emissions in ATTO than in the boreal zone (see Fig. S10), in MEGAN2.1, which is likely related to the fraction of the emissions which are light sensitive [see 4].

While it is fairly clear that the modelled OA mass is governed by biogenic local emissions for SMEAR-II (see Fig. S8), Fig. S13 shows that this is less dominant in the models at ATTO. In particular, ECHAM-SALSA seems to have a much clearer relationship if years 2015 and 2016 are excluded, but in these years the OA mass concentrations do not reduce in spite of almost zero biogenic emissions. Unfortunately, we do not have the output data to conclude what is happening in this model, but one theory could be that increases in forest fires are both giving lower BVOC emission and at the same time increasing emissions of biomass burning organic aerosol.

NorESM has a fairly strong relationship between emissions and OA at ATTO, while EC-Earth has a weak one and UKESM has a negative correlation for monoterpene (isoprene does not form SOA in UKESM). This pinpoints that the lack of relationship between temperature and OA in UKESM for ATTO originates not from the emissions side, but rather from the conversion of BVOCs to OA. Again, we cannot conclude, but one explanation for this could be a depletion of oxidants with high emissions

which would efficiently reduce the production of OA within the boundary layer.

Our aim was to limit the analysis to the season where the contribution of BSOA would be strongest. Figure S16 shows that the correlation between BVOC emissions and OA mass concentration was strongest around the selected time period.

S5 Regressions

S5.1 Regression coefficients for temperature to OA and OA to N_x

variable	data source	Fit	a	b	c	R^2	r^2
OA	UKESM	$a \cdot \exp(bx)$	5.86E-02 ± 1.97E-02	0.21 ± 0.35		0.58	0.79
OA	Observations	$a \cdot \exp(bx)$	0.12 ± 0.02	0.19 ± 0.30		0.45	0.73
OA	NorESM	$a \cdot \exp(bx)$	8.97E-02 ± 1.69E-02	0.18 ± 0.28		0.58	0.71
OA	ECHAM-SALSA	$a \cdot \exp(bx)$	1.71E-02 ± 2.22E-02	0.23 ± 0.39		0.53	0.71
OA	EC-Earth	$a \cdot \exp(bx)$	7.41E-02 ± 2.04E-02	0.20 ± 0.33		0.43	0.67
N50	UKESM	$ax+b$	110.5 ± 10.5	72.2 ± 34.4		0.62	0.81
N50	UKESM	$a+b\ln(c+x)$	-121.9 ± 165.6	400.1 ± 71.0	1.12 ± 0.62	0.7	0.81
N50	Observations	$ax+b$	274.8 ± 28.7	461.7 ± 96.3		0.01	0.51
N50	Observations	$a+b\ln(c+x)$	974.8 ± 94.1	393.5 ± 65.9	-0.25 ± 0.18	0.33	0.51
N50	NorESM	$ax+b$	703.5 ± 65.2	548.1 ± 155.9		0.82	0.91
N50	NorESM	$a+b\ln(c+x)$	-3486.3 ± 1452.9	3596.9 ± 552.7	2.73 ± 0.76	0.86	0.91
N50	ECHAM-SALSA	$ax+b$	325.2 ± 32.7	235.5 ± 49.9		0.24	0.62
N50	ECHAM-SALSA	$a+b\ln(c+x)$	535.5 ± 127.2	384.7 ± 92.8	0.30 ± 0.30	0.44	0.62
N50	EC-Earth	$ax+b$	141.2 ± 14.0	176.7 ± 34.1		0.61	0.81
N50	EC-Earth	$a+b\ln(c+x)$	180.7 ± 132.8	345.1 ± 67.7	0.49 ± 0.47	0.69	0.81
N200	UKESM	$ax+b$	44.1 ± 4.0	22.3 ± 13.3		0.95	0.98
N200	Observations	$ax+b$	53.6 ± 4.9	-11.5 ± 17.0		0.89	0.95
N200	NorESM	$ax+b$	29.6 ± 2.8	-0.32 ± 6.59		0.79	0.89
N200	ECHAM-SALSA	$ax+b$	35.5 ± 3.3	-0.98 ± 5.17		0.81	0.9
N200	EC-Earth	$ax+b$	42.3 ± 4.0	13.8 ± 9.9		0.91	0.96
N100	UKESM	$ax+b$	87.7 ± 8.3	68.6 ± 27.1		0.71	0.85
N100	UKESM	$a+b\ln(c+x)$	-242.8 ± 173.3	388.4 ± 68.6	1.75 ± 0.76	0.76	0.85
N100	Observations	$ax+b$	176.5 ± 16.6	85.9 ± 57.2		0.69	0.85
N100	Observations	$a+b\ln(c+x)$	-483.6 ± 302.5	760.3 ± 122.2	1.56 ± 0.67	0.77	0.85
N100	NorESM	$ax+b$	312.3 ± 28.5	-62.4 ± 68.6		0.94	0.97
N100	ECHAM-SALSA	$ax+b$	158.5 ± 14.8	22.9 ± 23.2		0.74	0.87
N100	ECHAM-SALSA	$a+b\ln(c+x)$	-360.8 ± 251.8	518.6 ± 112.0	1.94 ± 0.73	0.78	0.87
N100	EC-Earth	$ax+b$	93.9 ± 9.0	59.5 ± 22.2		0.82	0.91
N100	EC-Earth	$a+b\ln(c+x)$	-291.5 ± 186.4	401.2 ± 75.2	1.99 ± 0.79	0.85	0.91

Table S1. SMEAR-II: Overview over regression coefficients and properties. The parameters are presented with ± one standard deviation.

variable	data source	Fit	a	b	c	R^2	r^2
OA	UKESM	$a \cdot \exp(bx)$	2.38E-02 ± 3.00E-02	0.16 ± 0.91		-0.81	0.08
OA	Observations	$a \cdot \exp(bx)$	1.09E-06 ± 8.21E-02	0.52 ± 2.10		0.13	0.59
OA	NorESM	$a \cdot \exp(bx)$	1.14E-03 ± 5.05E-02	0.33 ± 1.27		0.33	0.69
OA	ECHAM-SALSA	$a \cdot \exp(bx)$	4.23E-03 ± 3.31E-02	0.20 ± 0.94		-0.08	0.44
OA	EC-Earth	$a \cdot \exp(bx)$	2.06E-03 ± 4.30E-02	0.27 ± 1.07		0.09	0.46
N50	UKESM	$ax+b$	112.7 ± 18.9	123.3 ± 30.6		-0.28	0.36
N50	Observations	$ax+b$	280.0 ± 42.9	67.6 ± 39.8		0.24	0.62
N50	NorESM	$ax+b$	357.7 ± 50.5	1623.0 ± 39.5		0.82	0.91
N50	ECHAM-SALSA	$ax+b$	172.6 ± 26.7	185.2 ± 53.6		0.19	0.59
N50	EC-Earth	$ax+b$	76.7 ± 11.1	178.9 ± 24.2		0.61	0.8
N200	UKESM	$ax+b$	48.1 ± 6.7	9.14 ± 10.95		0.95	0.98
N200	Observations	$ax+b$	69.3 ± 11.0	-7.02 ± 10.17		-0.01	0.5
N200	NorESM	$ax+b$	28.2 ± 3.9	-59.1 ± 3.1		0.93	0.97
N200	ECHAM-SALSA	$ax+b$	25.0 ± 3.5	-9.62 ± 7.21		0.87	0.94
N200	EC-Earth	$ax+b$	43.7 ± 6.1	-12.0 ± 13.3		0.94	0.97
N100	UKESM	$ax+b$	79.5 ± 11.9	118.3 ± 19.5		0.38	0.69
N100	Observations	$ax+b$	189.3 ± 28.9	20.4 ± 26.9		0.26	0.63
N100	NorESM	$ax+b$	292.4 ± 40.5	289.9 ± 31.8		0.97	0.98
N100	ECHAM-SALSA	$ax+b$	111.8 ± 17.8	-26.4 ± 35.4		0.02	0.51
N100	EC-Earth	$ax+b$	62.6 ± 8.8	63.0 ± 19.2		0.85	0.93

Table S2. ATTO: Overview over regression coefficients and properties. The parameters are presented with ± one standard deviation.

variable	data source	Fit	a	b	c	R^2	r^2
OA	UKESM	$a \cdot \exp(bx)$	3755.61 ± 0.07	-0.24 ± 1.99		-0.94	-0.04
OA	Observations	$a \cdot \exp(bx)$	2.98E-07 ± 1.28E-01	0.56 ± 3.25		0.16	0.66
OA	NorESM	$a \cdot \exp(bx)$	6.21E-04 ± 8.11E-02	0.36 ± 2.00		0.15	0.58
OA	ECHAM-SALSA	$a \cdot \exp(bx)$	7.87E-06 ± 1.02E-01	0.47 ± 2.67		0.38	0.68
OA	EC-Earth	$a \cdot \exp(bx)$	5.16E-03 ± 5.06E-02	0.24 ± 1.24		0.49	0.75

Table S3. ATTO without years 2015/2016: Overview over regression coefficients and properties. The parameters are presented with ± one standard deviation.

S6 Regression with linear lines for OA to N_x

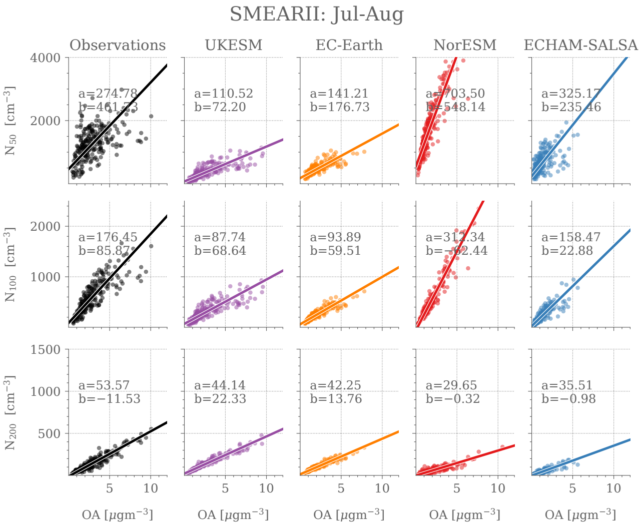

Figure S17. SMEAR-II: Same as Fig. 3, but with linear fits. The relationship between daily median OA and the number concentration of particles larger than 50 nm (N_{50}), 100 nm (N_{100}) and 200 nm (N_{200}). The lines show the orthogonal distance regression to a linear function ($ax+b$) (see Methods for details).

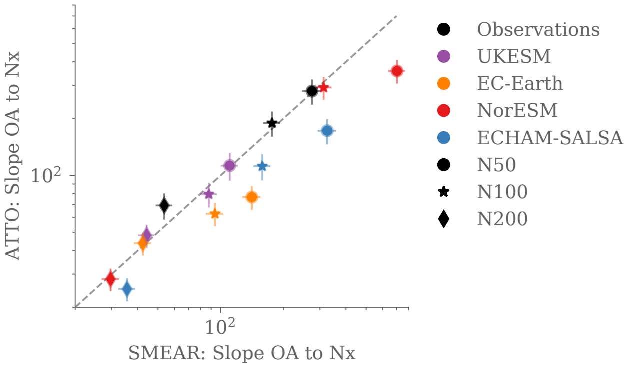

Figure S18. Comparison of the slope of the fits in Fig. S17 between SMEAR-II (x-axis) and ATTO (y-axis), i.e. the slope of the linear regression (a in $ax+b$) of the relationship between daily median OA and the number concentration of particles larger than 50 nm (N_{50}), 100 nm (N_{100}) and 200 nm (N_{200}). The uncertainty is shown with $\pm\sigma$.

S6.1 Residuals for fitted regressions

S6.1.1 Temperature to OA

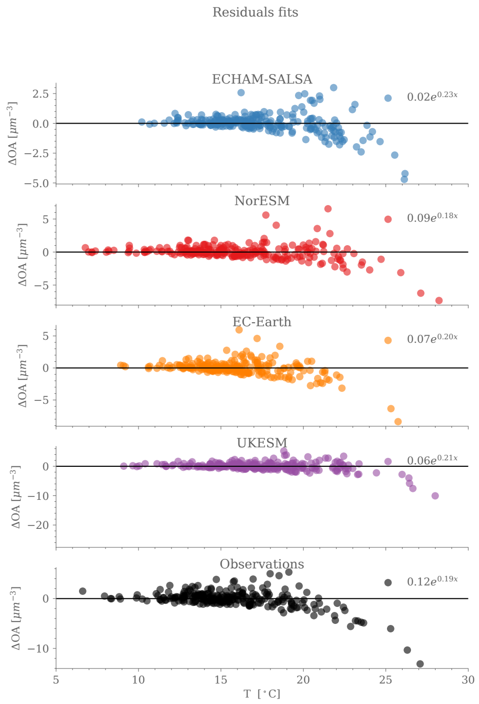

Figure S19. SMEAR-II: Residuals for relationship between temperature and organic aerosol mass concentration in models and observations shown in Fig. 2. The regressions are given in the legend.

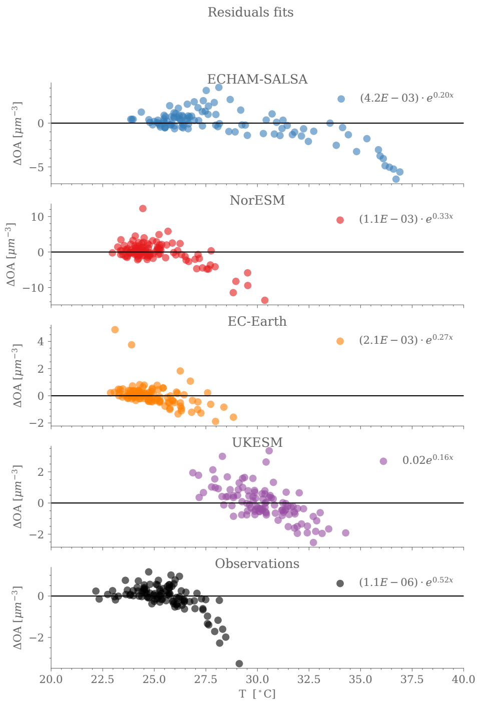

Figure S20. ATTO: Residuals for Relationship between temperature and organic aerosol mass in models and observations shown in Fig. 2. The regressions are given in the legend.

S6.1.2 OA mass to N_x

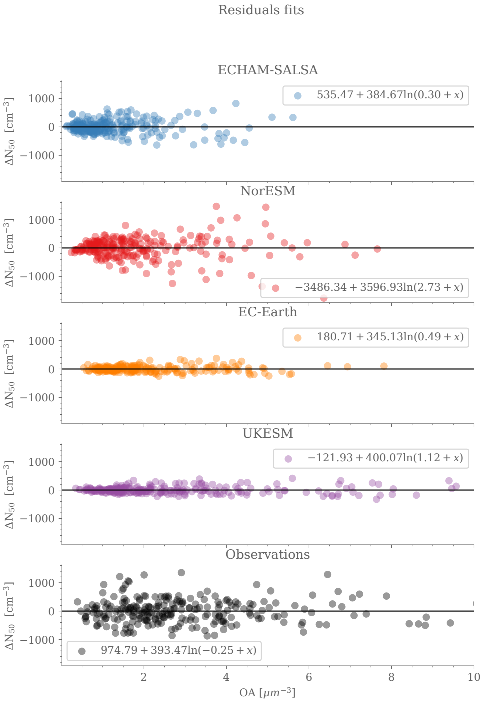

Figure S21. SMEAR-II: Residuals for regression between organic aerosol mass (OA) and N_{50} in models and observations shown in Fig. 3. The regressions are given in the legend.

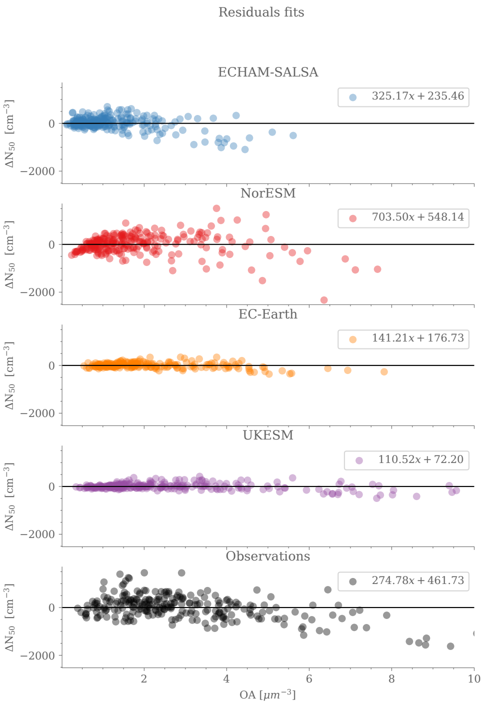

Figure S22. SMEAR-II: Residuals for regression between organic aerosol mass (OA) and N_{50} in models and observations shown in Fig. S17. The regressions are given in the legend.

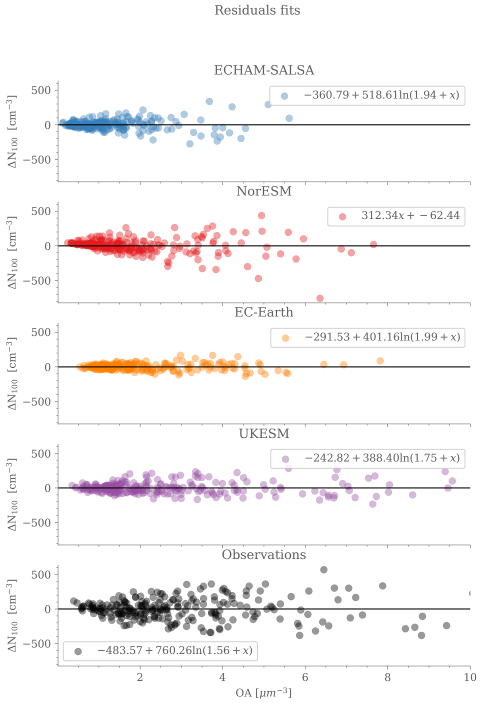

Figure S23. SMEAR-II: Residuals for regression between organic aerosol mass (OA) and N_{100} in models and observations shown in Fig. 3. The regressions are given in the legend.

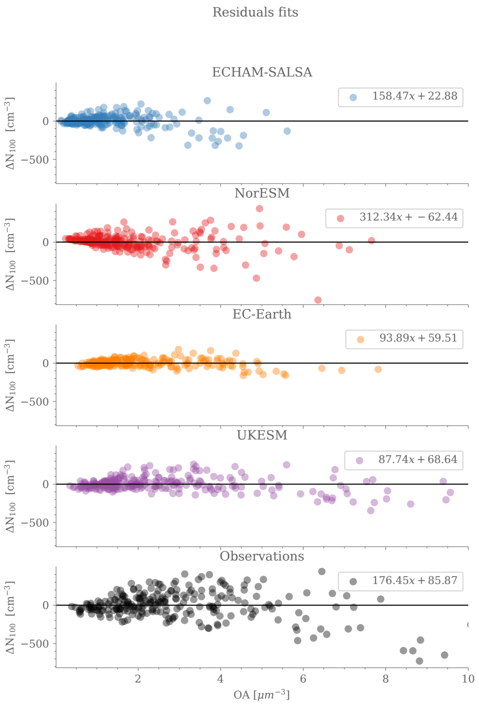

Figure S24. SMEAR-II: Residuals for regression between organic aerosol mass (OA) and N_{100} in models and observations shown in Fig. S17. The regressions are given in the legend.

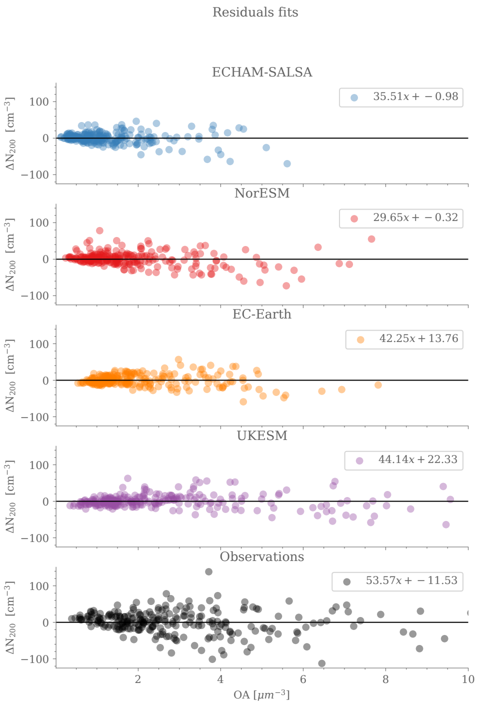

Figure S25. SMEAR-II: Residuals for regression between organic aerosol mass (OA) and N_{200} in models and observations shown in Fig. 3 and S17. The regressions are given in the legend.

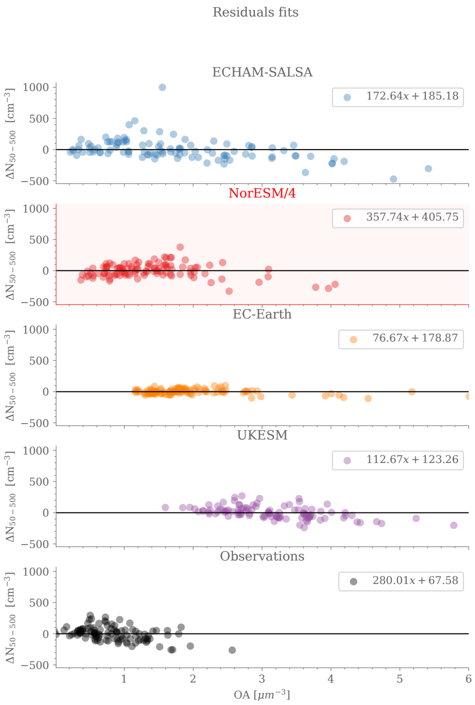

Figure S26. ATTO: Residuals for regression between organic aerosol mass (OA) and N_{50-500} in models and observations shown in Fig. 3 and S17. The regressions are given in the legend. Note that the NorESM values are divided by 4 (both x-values and y-values).

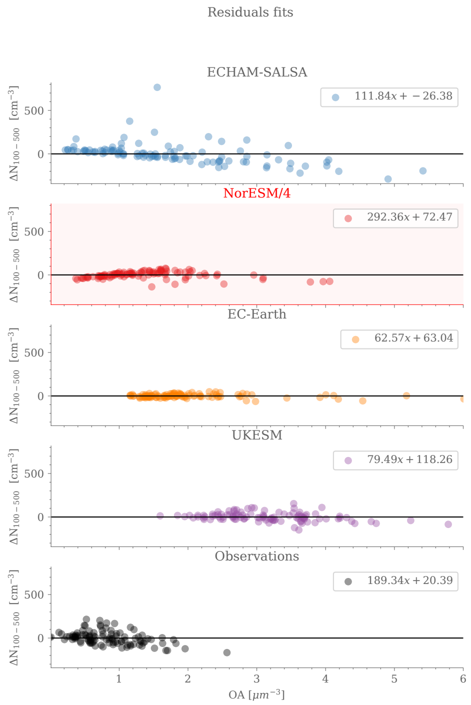

Figure S27. ATTO: Residuals for regression between organic aerosol mass (OA) and $N_{100-500}$ in models and observations shown in Fig. 3 and S17. The regressions are given in the legend. Note that the NorESM values are divided by four (both x-values and y-values).

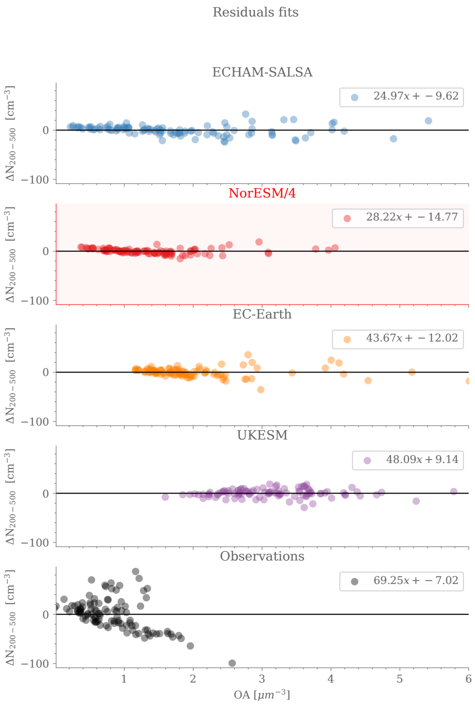

Figure S28. ATTO: Residuals for regression between organic aerosol mass (OA) and $N_{200-500}$ in models and observations shown in Fig. 3 and S17. The regressions are given in the legend. Note that the NorESM values are divided by 4 (both x-values and y-values).

S7 Cloud properties

S7.1 Overview cloud properties through the year at ATTO

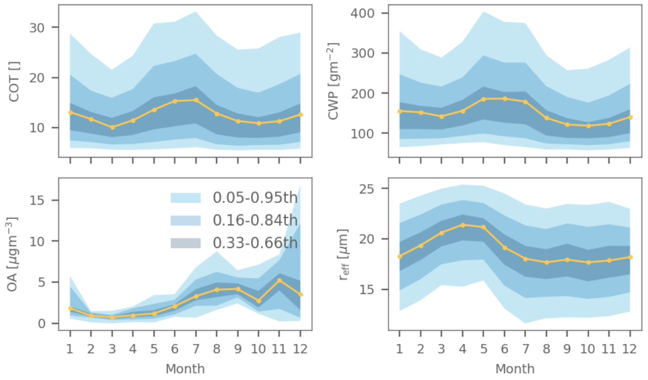

Figure S29. ATTO: Cloud properties and variability through the year as calculated from daytime mean values in the area of consideration and the OA mass concentration measured at the site.

S7.2 Cloud impacts of high versus low CCN (N_{50} and N_{100})

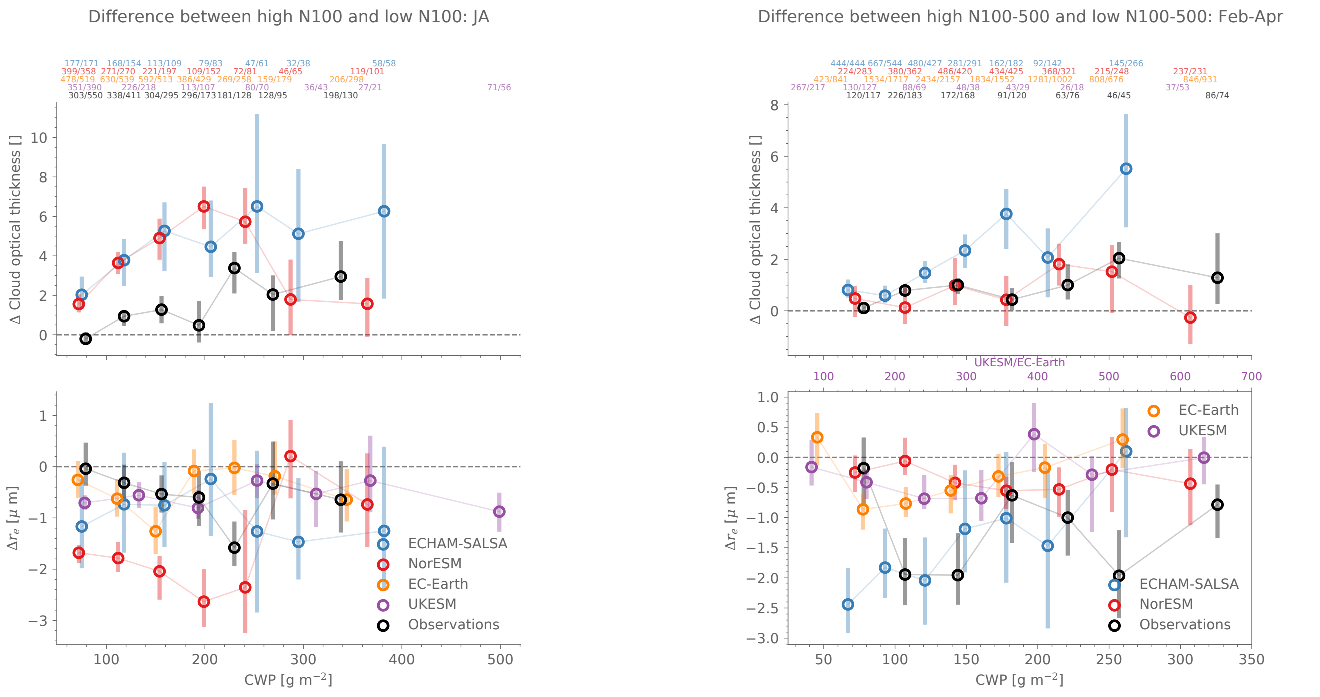

(a) SMEAR-II: Jul–Aug.

(b) ATTO: Feb–Apr.

Figure S30. Difference in median cloud optical thickness (top) and cloud droplet effective radius (bottom) between high N_{100} (above 67th percentile) days and low N_{100} (below 34th percentile) days. The uncertainties marked by the bars and are the 2.5th to 97.5th percentile of the median calculated by bootstrapping and the percentile method (see Methods section). The numbers at the top signify the number of data points in low/high OA in each CWP bin.

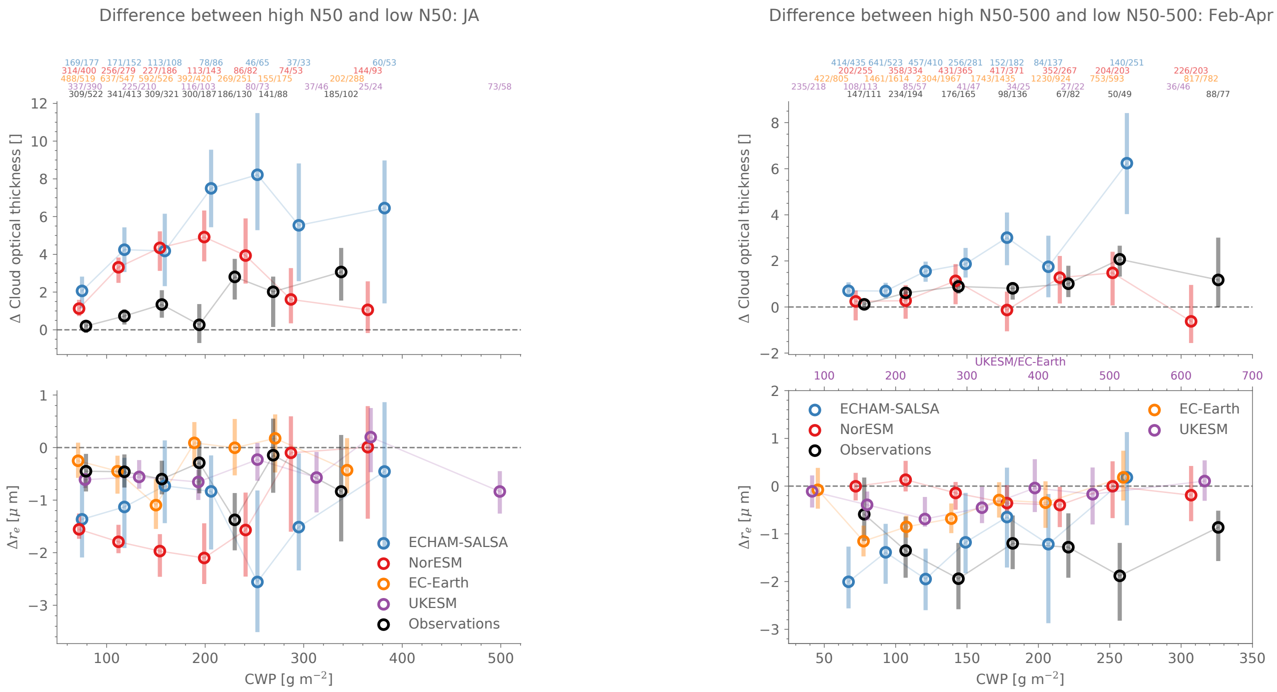

(a) SMEAR-II: Jul–Aug.

(b) ATTO: Feb–Apr.

Figure S31. Difference in median cloud optical thickness (top) and cloud droplet effective radius (bottom) between high N_{50} (above 67th percentile) days and low N_{50} (below 34th percentile) days. The uncertainties marked by the bars and are the 2.5th to 97.5th percentile of the median calculated by bootstrapping and the percentile method (see Methods section). The numbers at the top signify the number of data points in low/high OA in each CWP bin.

S7.3 Distributions of cloud properties

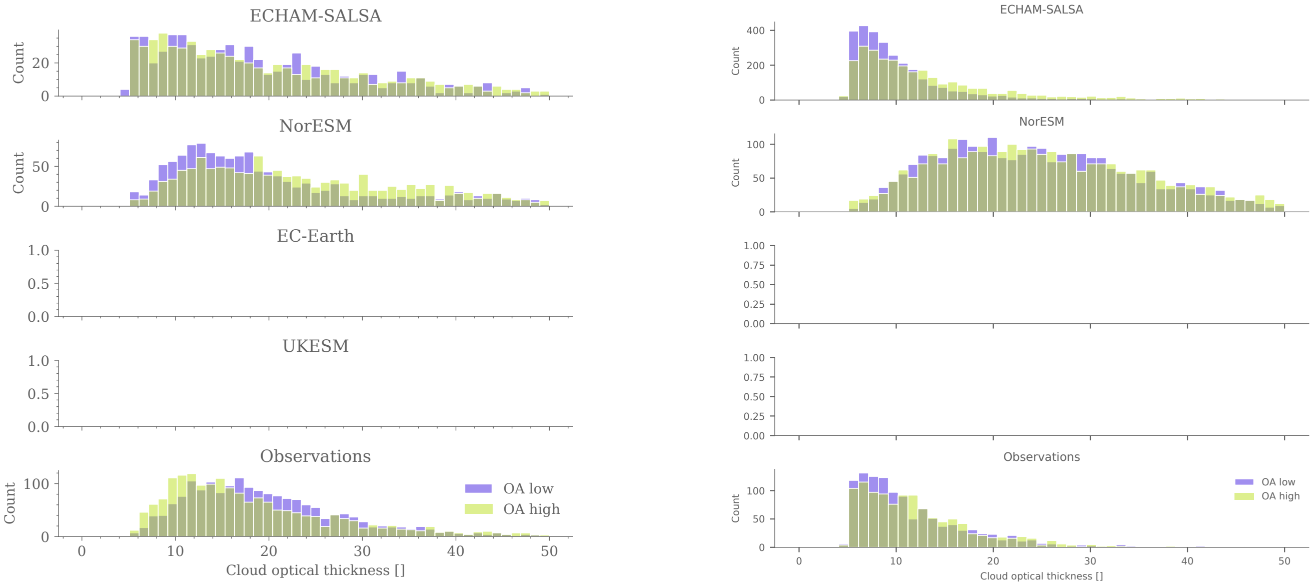

(a) SMEAR-II

(b) ATTO

Figure S32. Distribution of COT at SMEAR-II in July–August (left) and ATTO in February–April (right).

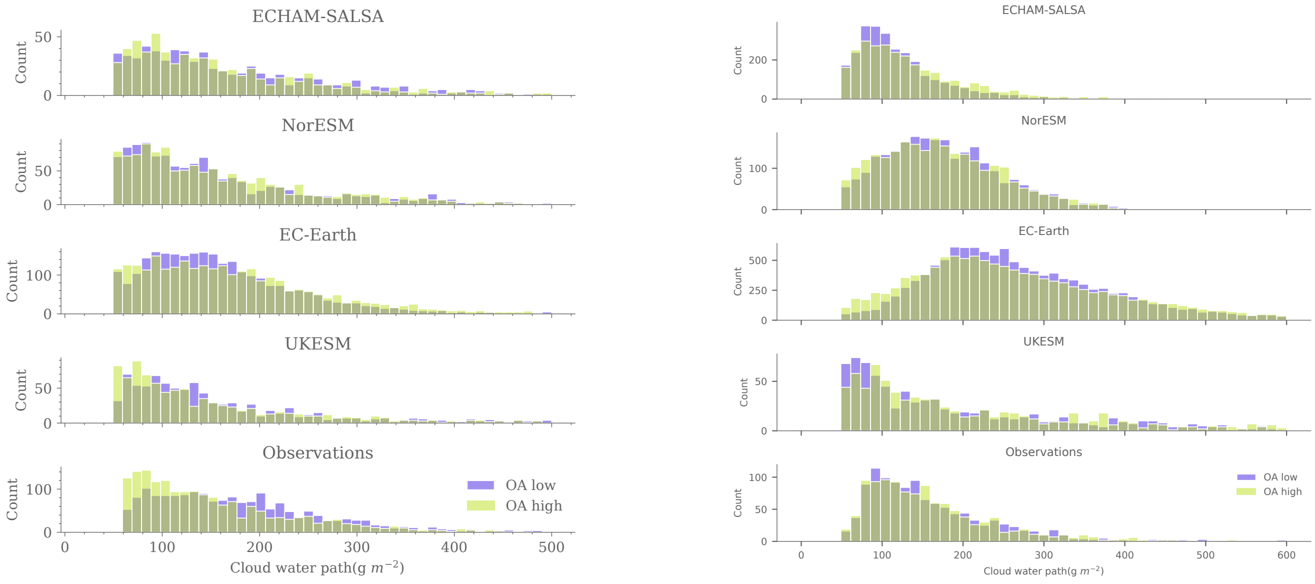

(a) SMEAR-II

(b) ATTO

Figure S33. Distribution of CWP at SMEAR-II in July–August (left) and ATTO in February–April (right).

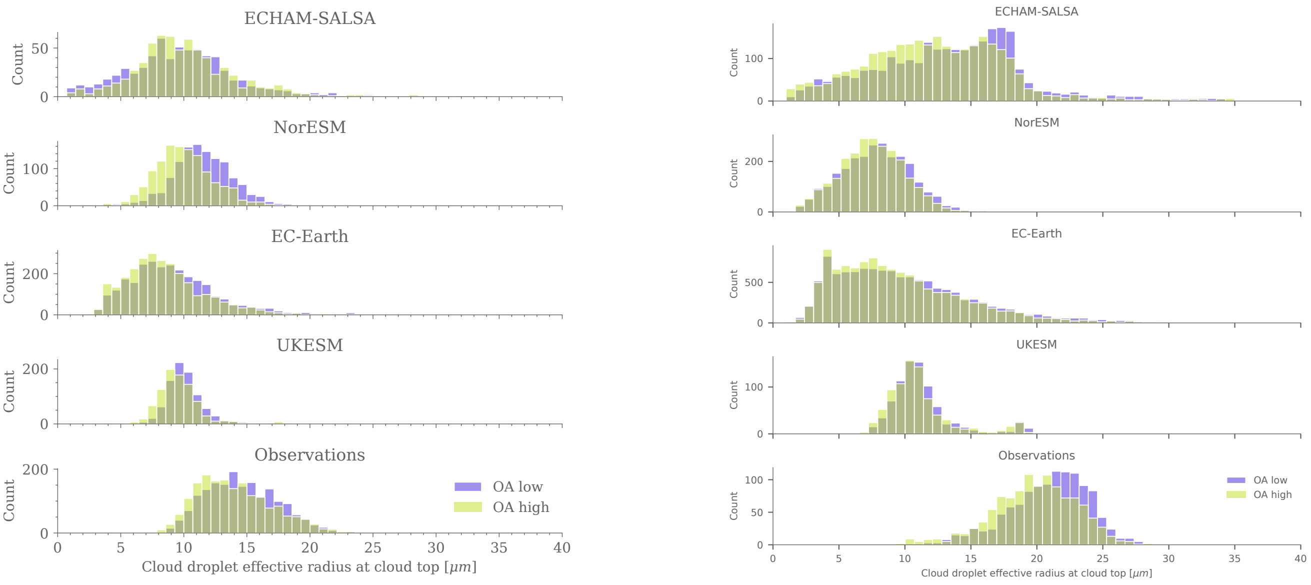

(a) SMEAR-II **(b)** ATTO

Figure S34. Distribution of r_{eff} at SMEAR-II in July–August (left) and ATTO in February–April(right).

S8 Detailed discussion on regression coefficients

S8.1 Temperature to organic aerosol mass

As mentioned in the main text, the relationship between temperature and OA mass is best described by an exponential function of the form $OA = \alpha \exp(\beta T)$ (see residuals in Figure S19 and S20). This is in line with the expected exponential relationship between temperature and emissions of BVOCs [4, 5] and with the assumption of the availability of BVOC precursors being a major factor controlling SOA formation at the two sites rather than e.g. temperature-dependent volatility of the oxidation products (see also [1]). In the equation above, α incorporates factors for the baseline emission strength of BVOCs, mass yield of SOA, and the loss rates of SOA. This allows for evaluating the models with respect to both the SOA yields/baseline emissions/losses (combined in the α-term) and the strength of the temperature response (the β term, i.e. the slopes of the fits as they appear in linear-logarithmic space in Fig. 2). See Tab. S1–S2 for an overview of the regressions and their properties.

At SMEAR-II, the models all underestimate OA mass concentration (see also Fig. S37 and S39), however, the exponential coefficient, β, (the slope of the line in Fig. 2) is surprisingly well represented in the models. Essentially this means that if the model SOA yields were increased with some factor, we would have a very good fit, all else equal. For ATTO, all models tend to overestimate the total OA mass concentrations: NorESM median is approximately an order of magnitude too high, UKESM and EC-Earth have too high concentrations and too narrow distributions, while ECHAM-SALSA might be said to be slightly better in terms of capturing the variability (wider distribution)(see also Fig. S49). NorESM and EC-Earth are close to representing the β term accurately, though still with an underestimation.

ECHAM-SALSA and UKESM both have unrealistically high temperatures (see histogram on the top of Fig. 2, right panel), which could indicate that the models struggle with capturing the tropical forest environment and might not get the latent to sensible heat fluxes right. This is, however, unlikely to be the reason for the low increase in OA mass with temperature, because for EC-Earth the emissions are pre-calculated with LPJ-Guess and for ECHAM-SALSA, the soil moisture is not used in the emission calculation.

Some underestimation of OA mass in the models would also be expected if there is a strong vertical gradient in the OA concentration close to the surface and the measurements are done close to the ground (the case at SMEAR-II, where the inlet is at 4 meters), given the vertical level closest to the surface is usually around 100 meters. If this was a major influence, however, one might expect the underestimation of OA to be stronger during night, when the atmosphere is more stratified, but evaluation of the diurnal variation in NorESM in particular, shows this to not be the case (see Fig. S38).

S8.1.1 Yield tuning: An example with NorESM

One way one might consider improving the models in terms of OA mass, is to tune the yields for isoprene and monoterpene respectively, so that OA might increase at SMEAR-II and decrease at ATTO (see Results section). However, a highly simplified investigation shows that this is unlikely to succeed for NorESM. In Fig. S35, the initial mean distribution (daily mean values)

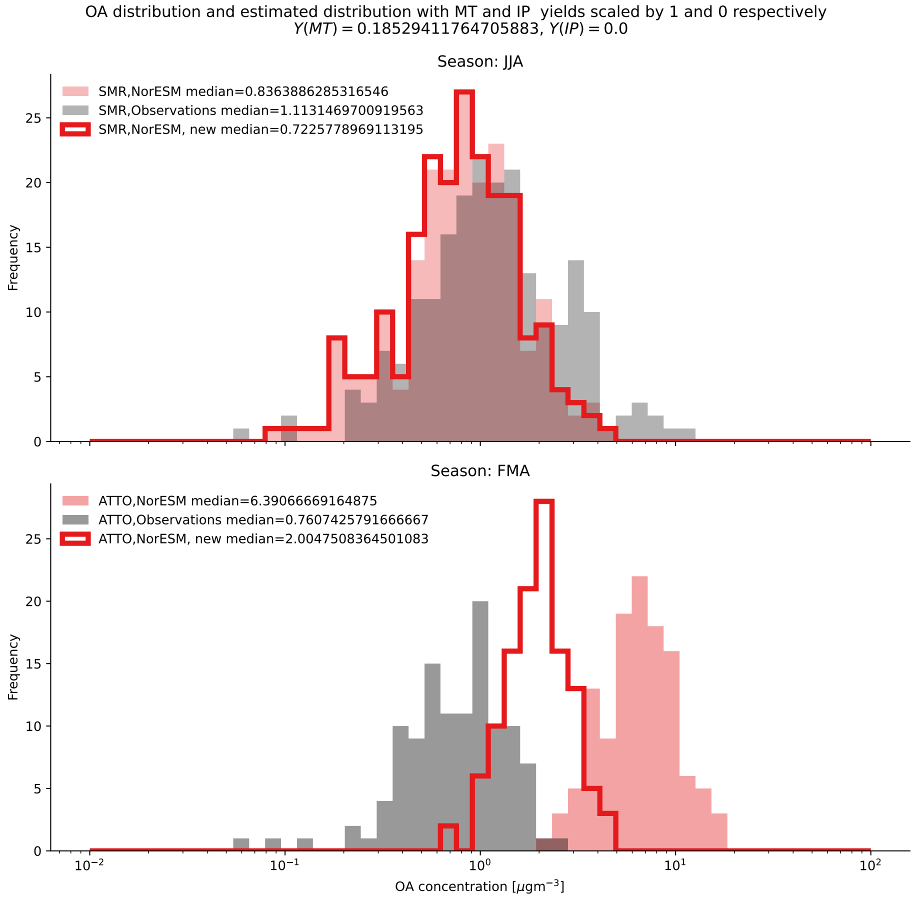

Figure S35. Distribution of daily mean values of OA mass at SMEAR-II (top) and ATTO (bottom) for observations and NorESM. The solid line is showing the distribution if the OA mass concentration is scaled down as estimated from a zero SOA yield for isoprene.

at SMEAR-II (top) and ATTO (bottom) are shown for the observed and NorESM. The line plot shows the distribution when subtracting the estimated contribution to OA from isoprene, assuming (1) all the OA is biogenic, (2) the OA from IP can be estimated as $OA_{IP} = OA_{tot} \cdot \frac{f_{IP}Y_{IP}}{f_{IP}Y_{IP}+f_{MT}Y_{MT}}$ where f_{IP} and f_{MT} are the mass fraction of IP and MT, respectively, to the total BVOC emissions (MT+IP), and Y_{IP} and Y_{MT} are the SOA mass yield of IP and MT, respectively. As can be seen, even for isoprene yields set to zero, there is a significant overestimation of OA mass at ATTO. This indicates that the issue cannot be solved by tuning the yields. There are several reasons why this might be. One could be that the high isoprene emissions in the Amazon are reducing the SOA production and that the model needs an interaction term in the yield calculation, as would be indicated by e.g. [6]. On the other hand, the flaw might very well also lie in the emissions where NorESM gives much higher values than the other models [7]) or the loss processes in the model.

S9 Basic aerosol evaluation: SMEAR-II

S9.1 Organic aerosol mass

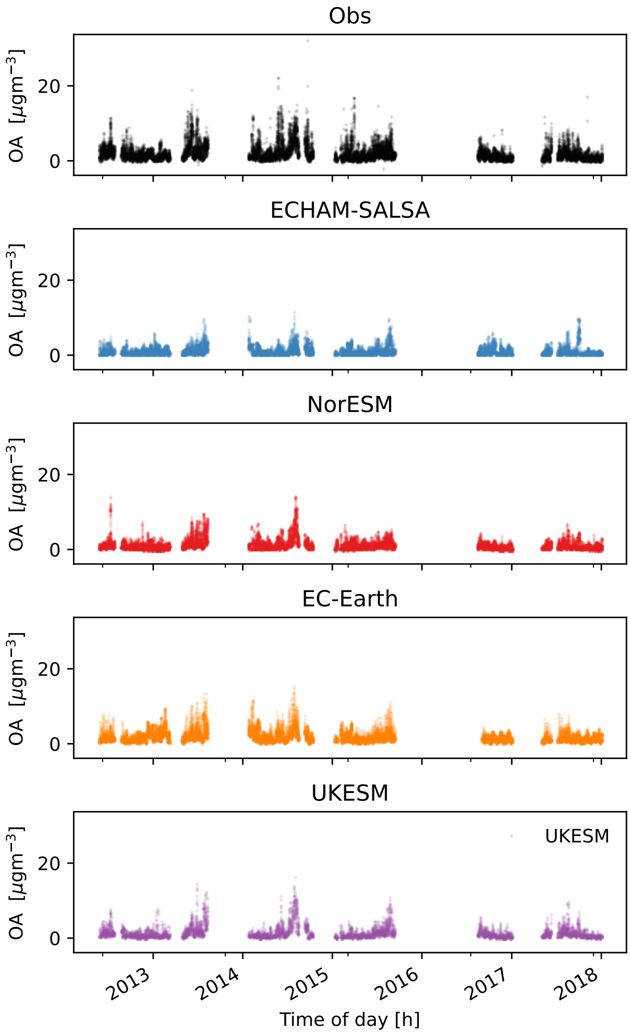

Figure S36. SMEAR-II: Time series of OA mass concentration from models and observations SMEAR-II. The modelled data is shown only when there is not missing data in the observations. The data is in hourly resolution for observations and all models except UKESM for which we had 3 hourly output.

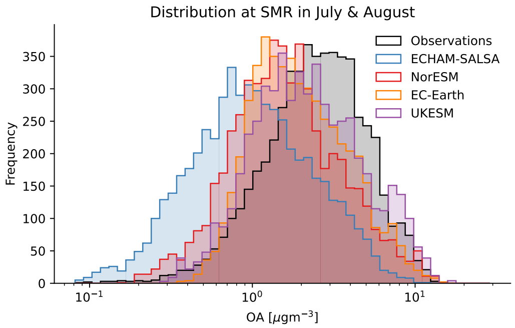

Figure S37. SMEAR-II: Distribution of OA mass concentration in models and observations at SMEAR-II in July and August. The data is in hourly resolution for observations and all models except UKESM for which we had 3 hourly output. UKESM values were repeated 3 times here to match the frequency of the other data sources (affects the frequency, but not the shape of the distribution).

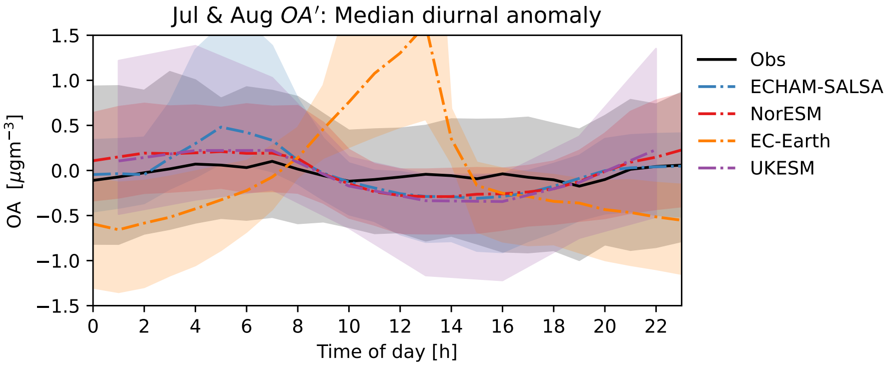

Figure S38. SMEAR-II: Diurnal variability of OA mass concentration at SMEAR-II. Lines signify median deviation from daily mean value and the shading signifies the 16th to 84th percentiles from data in hourly resolution. Model values included only if the corresponding time exists in the observations. The data is in hourly resolution for observations and all models except UKESM for which we had 3 hourly output.

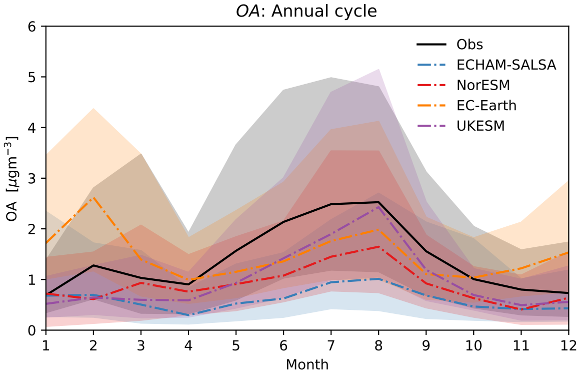

Figure S39. SMEAR-II: Monthly median OA mass concentration at SMEARII. Lines signify median as calculated from all available data and shading signifies the 16th to 84th percentiles from data in hourly resolution (except UKESM which is in 3 hourly resolution). Model values included only if the corresponding time exists in the observations. This computation method entails some more weight to years with more data available.

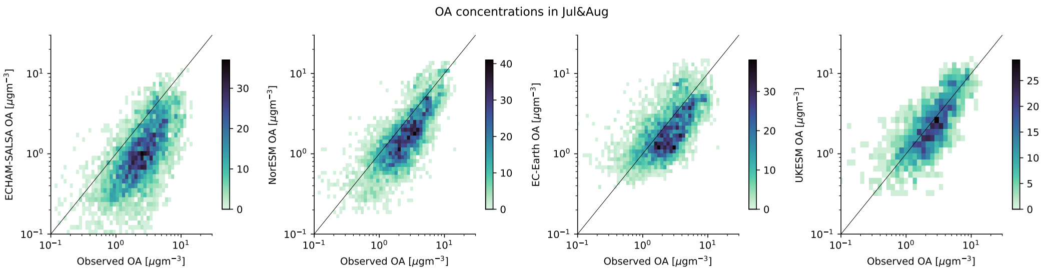

Figure S40. SMEAR-II: Distribution of modelled versus observed OA mass in July and August. The data is in hourly resolution for observations and all models except UKESM for which we had 3 hourly output.

S9.2 Aerosol concentrations: N50, N100 and N200

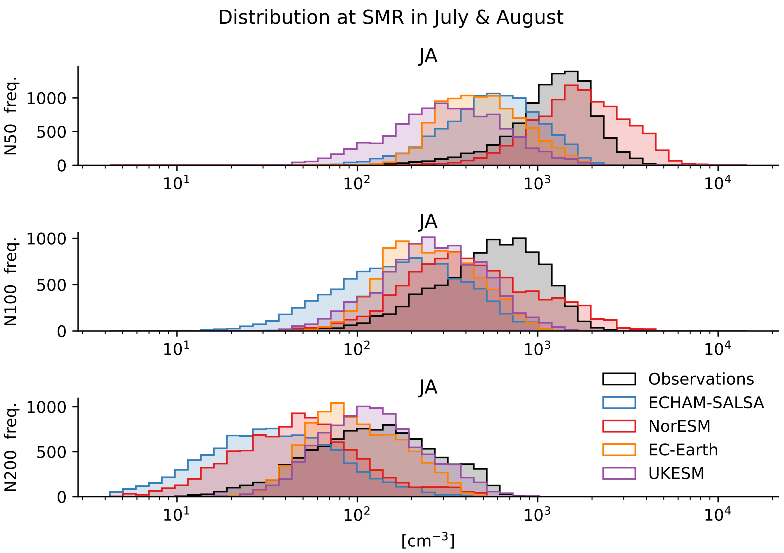

Figure S41. Distribution of N50, N100 and N200 in July–August from hourly data, except UKESM for which it is 3 hourly values. UKESM values were repeated 3 times here to match the frequency of the other data sources (affects the frequency, but not the shape of the distribution).

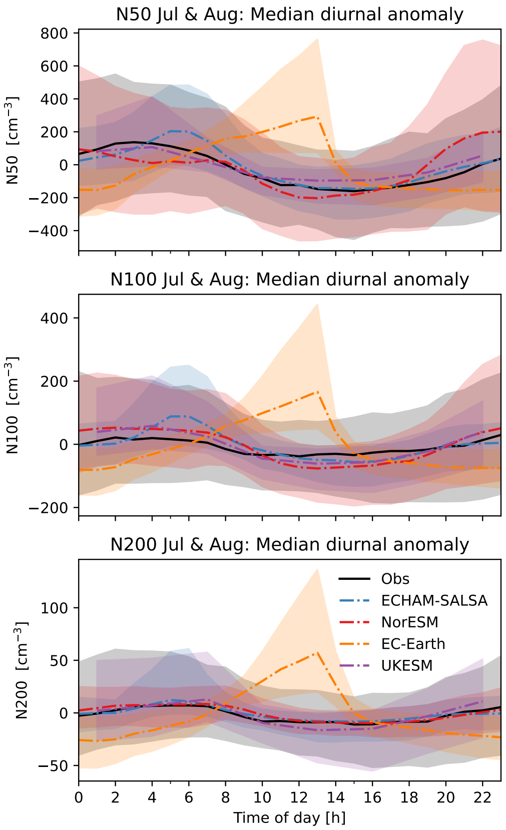

Figure S42. SMEAR-II: Diurnal variability of N_{50} (top), N_{100} (middle) and N_{200} (bottom) concentration at SMEAR-II. Lines signify median deviation from daily mean value and the shading signifies the 16th to 84th percentiles from data in hourly resolution. Model values included only if the corresponding time exists in the observations. The input is in hourly resolution, except for UKESM which has 3 hourly values.

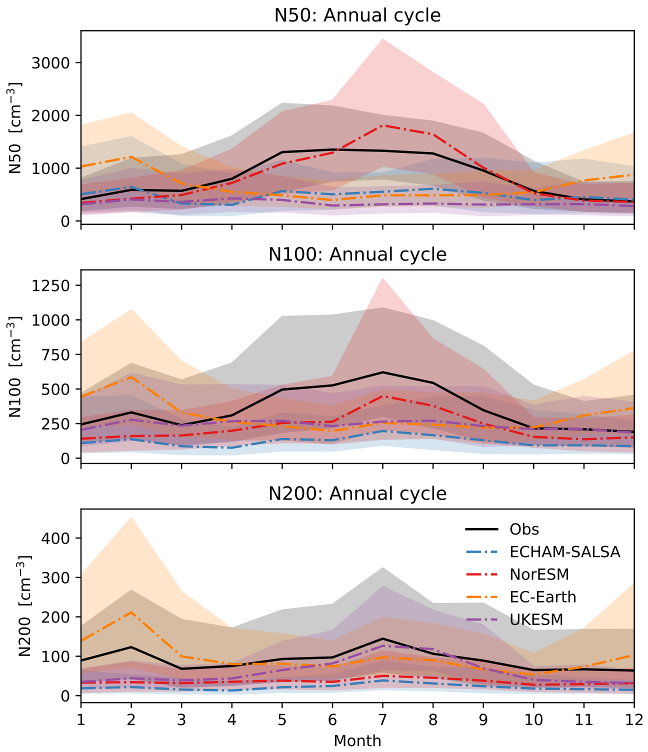

Figure S43. SMEAR-II: Monthly median N_{50} (top), N_{100} (middle) and N_{200} (bottom) concentration at SMEARII. Lines signify median values as calculated from all available data (hourly resolution for all sources except UKESM which has 3 hourly data) and shading signifies the 16th to 84th percentiles from data in hourly resolution. Model values included only if the corresponding time exists in the observations. This computation method entails some more weight to years with more data available.

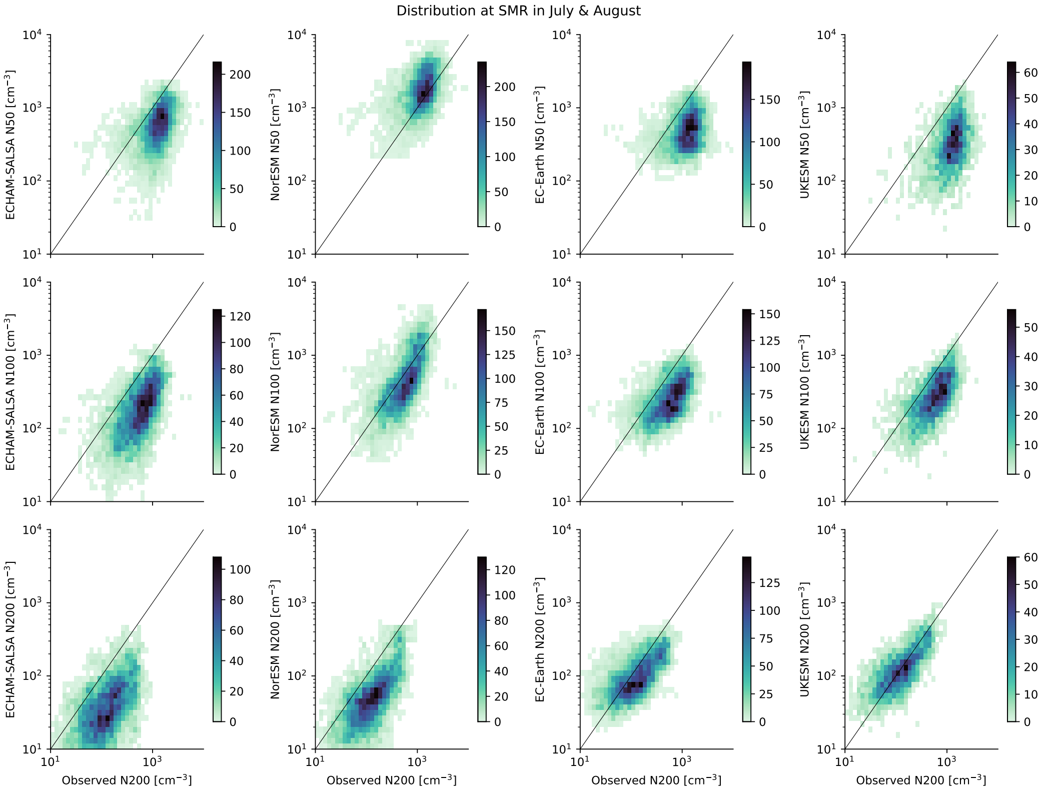

Figure S44. SMEAR-II: Distribution of observed (x-axis) versus modelled (y-axis) N_{50} (top row), N_{100} (middle row) and N_{200} (bottom row), in hourly resolution. The color signifies the frequency. The data is in hourly resolution for observations and all models except UKESM for which we had 3 hourly output.

S9.3 Time series N50, N100, N200

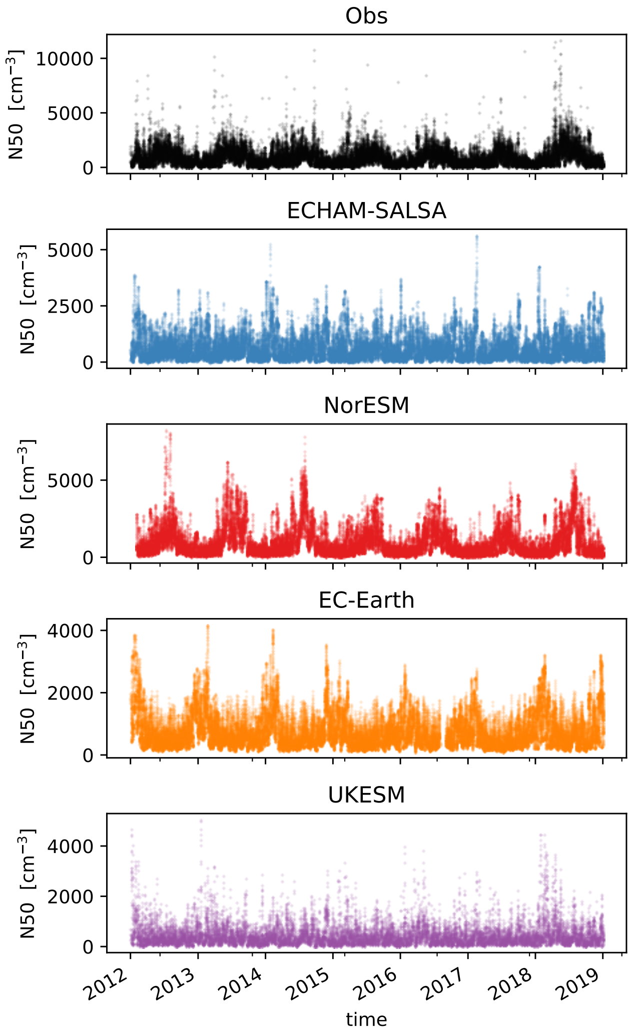

Figure S45. SMEAR-II: Time series of models and observations of N_{50}. The modelled data is shown only when there is not missing data in the observations. The data is in hourly resolution for observations and all models except UKESM for which we had 3 hourly output.

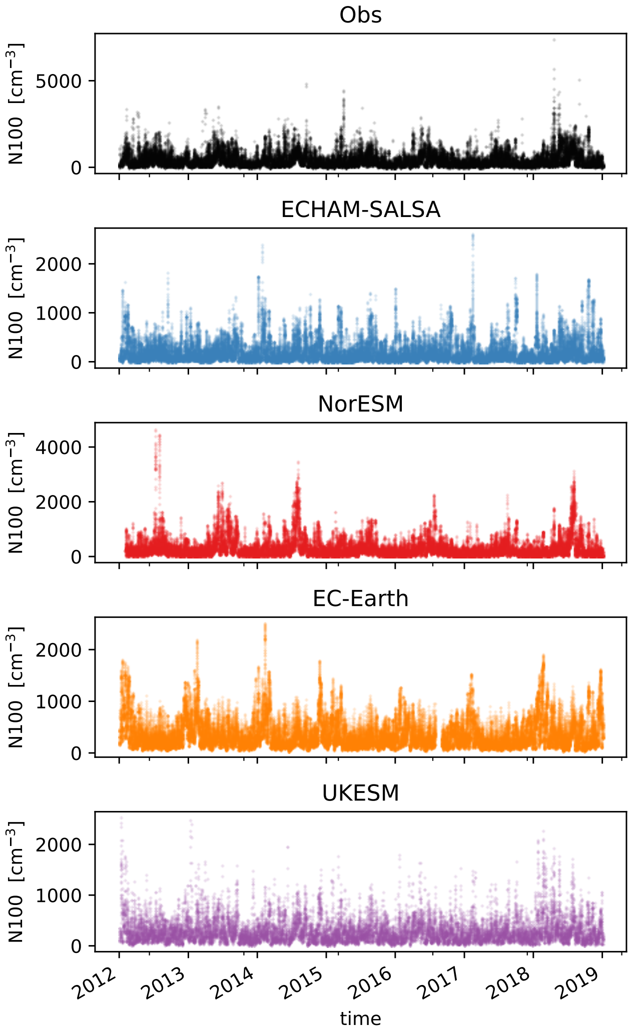

Figure S46. SMEAR-II: Time series of models and observations of N_{100}. The modelled data is shown only when there is not missing data in the observations. The data is in hourly resolution for observations and all models except UKESM for which we had 3 hourly output.

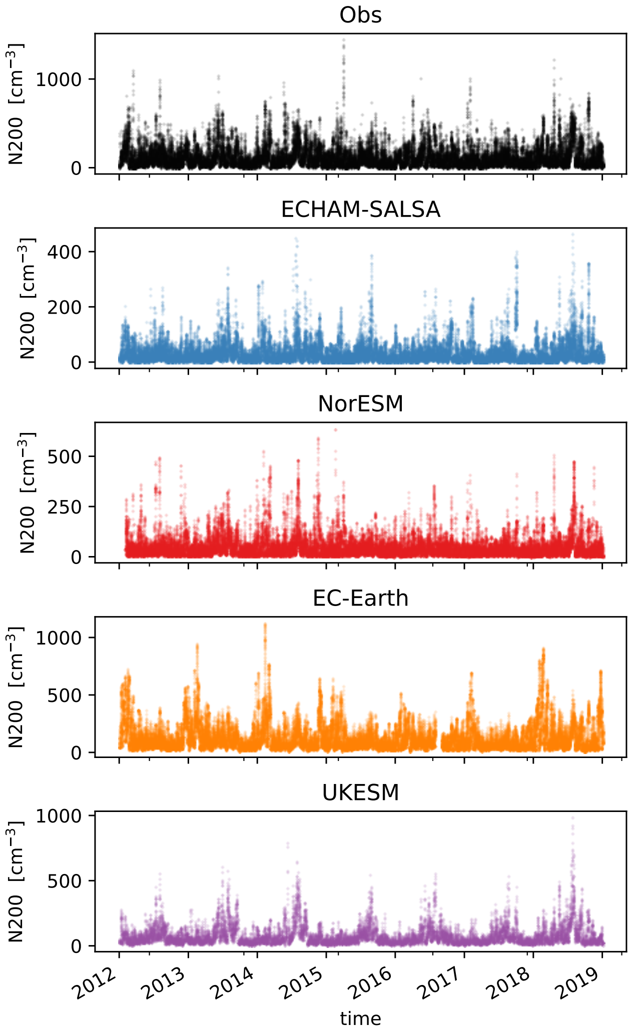

Figure S47. SMEAR-II: Time series of models and observations of N_{200}. The modelled data is shown only when there is not missing data in the observations. The data is in hourly resolution for observations and all models except UKESM for which we had 3 hourly output.

S10 Basic aerosol evaluation: ATTO

S10.1 Organic aerosol mass

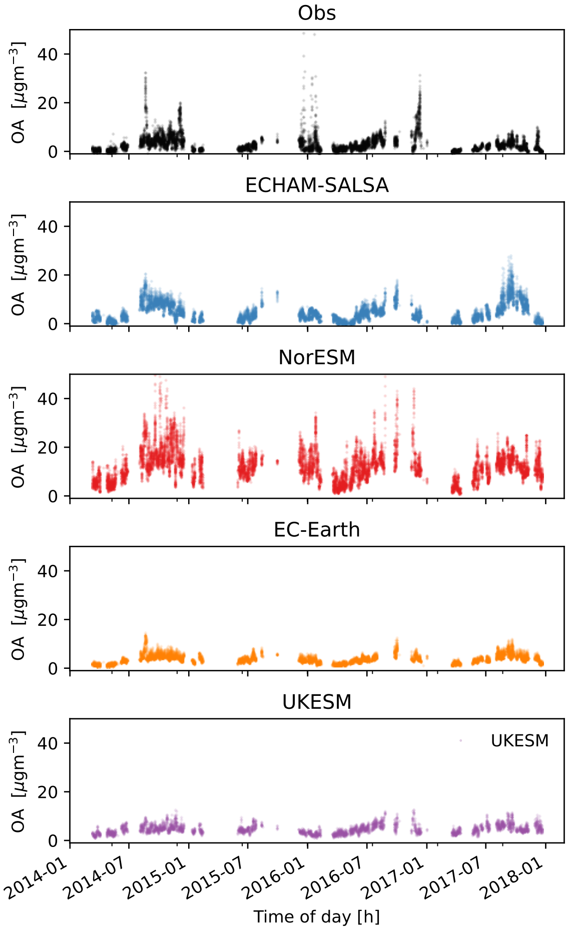

Figure S48. ATTO: Time series of OA mass concentration from models and observations ATTO. The modelled data is shown only when there is not missing data in the observations. The data is in hourly resolution for observations and all models except UKESM for which we had 3 hourly output.

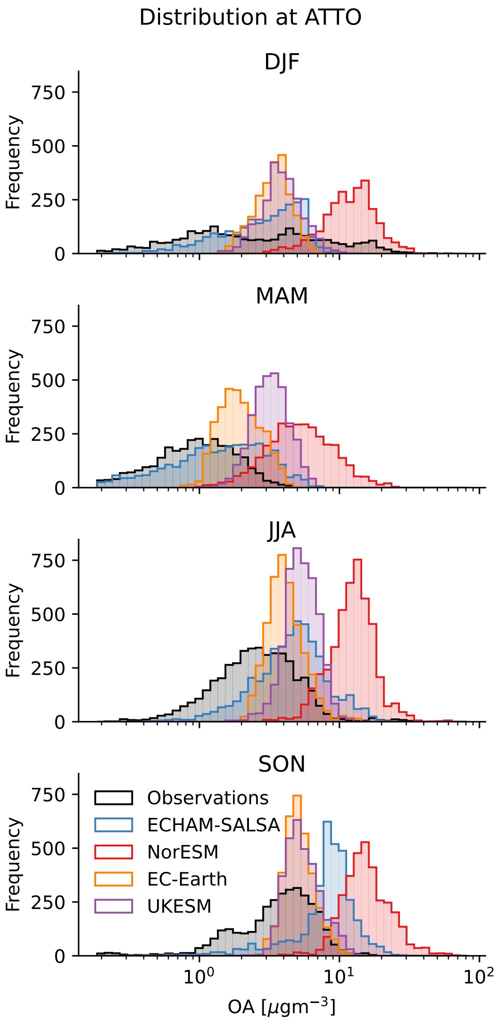

Figure S49. ATTO: Distribution of OA mass in different seasons from hourly resolution. The data is in hourly resolution for observations and all models except UKESM for which we had 3 hourly output. UKESM values are repeated for missing hours to match the frequency of the other data sources because UKESM only has 3 hourly output.

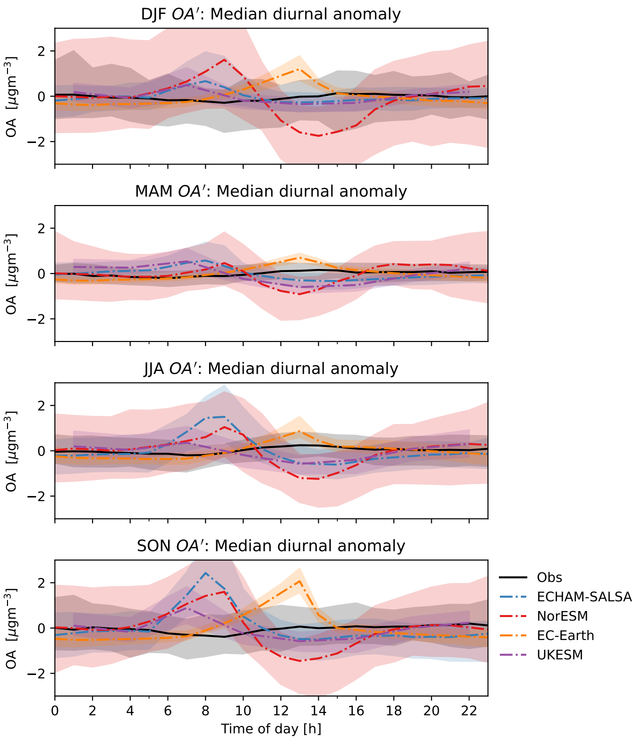

Figure S50. ATTO: Diurnal variability of OA mass concentration in different seasons. Lines signify median deviation from daily mean value and the shading signifies the 16th to 84th percentiles from data in hourly resolution, except UKESM which has 3 hourly values. Model values included only if the corresponding time exists in the observations.

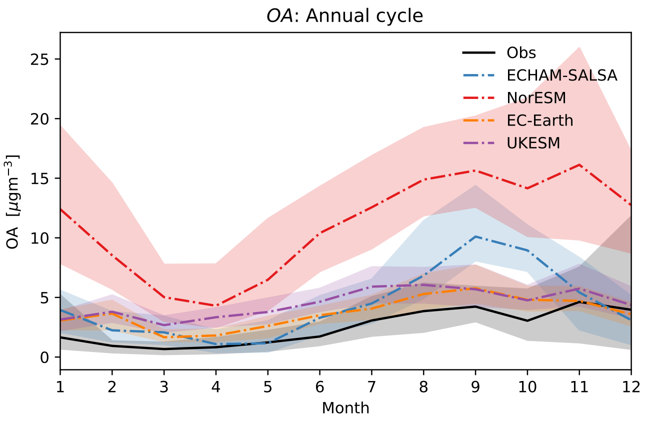

Figure S51. ATTO: Monthly median OA mass concentration. Lines signify median as calculated from all available data and shading signifies the 16th to 84th percentiles from data in hourly resolution, except UKESM which has 3 hourly values. Model values included only if the corresponding time exists in the observations. This computation method entails some more weight to years with more data available.

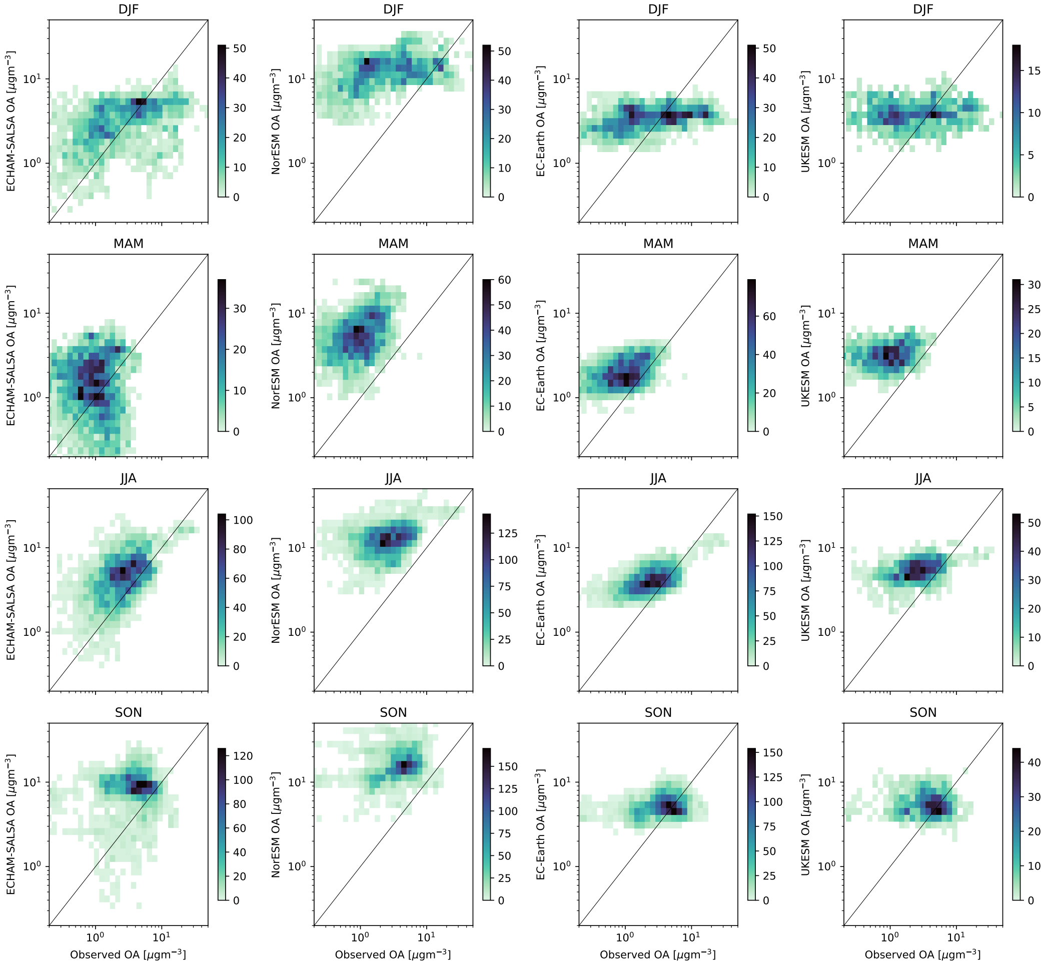

Figure S52. ATTO: Distribution of observed (x-axis) versus modelled (y-axis) OA mass in DJF (top row), MAM (second row), JJA (third row) and SON (bottom row). The color signifies the frequency. The data is in hourly resolution for observations and all models except UKESM for which we had 3 hourly output.

S10.2 Aerosol concentrations: N50, N100 and N200

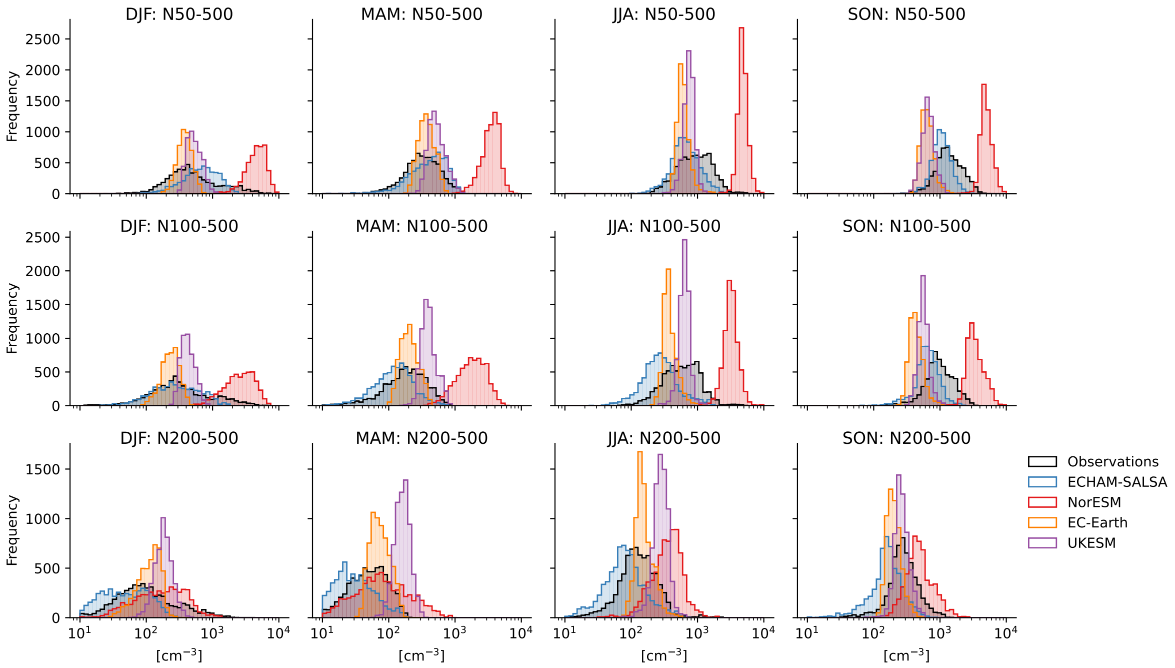

Figure S53. ATTO: Distribution of N_{50} (first row), N_{100} (second row) and N_{200} (third row) in DJF (first column), MAM (second column), JJA (third column) and SON (last column). The data is in hourly resolution for observations and all models except UKESM for which we had 3 hourly output. UKESM values were repeated 3 times here to match the frequency of the other data sources (affects the frequency, but not the shape of the distribution).

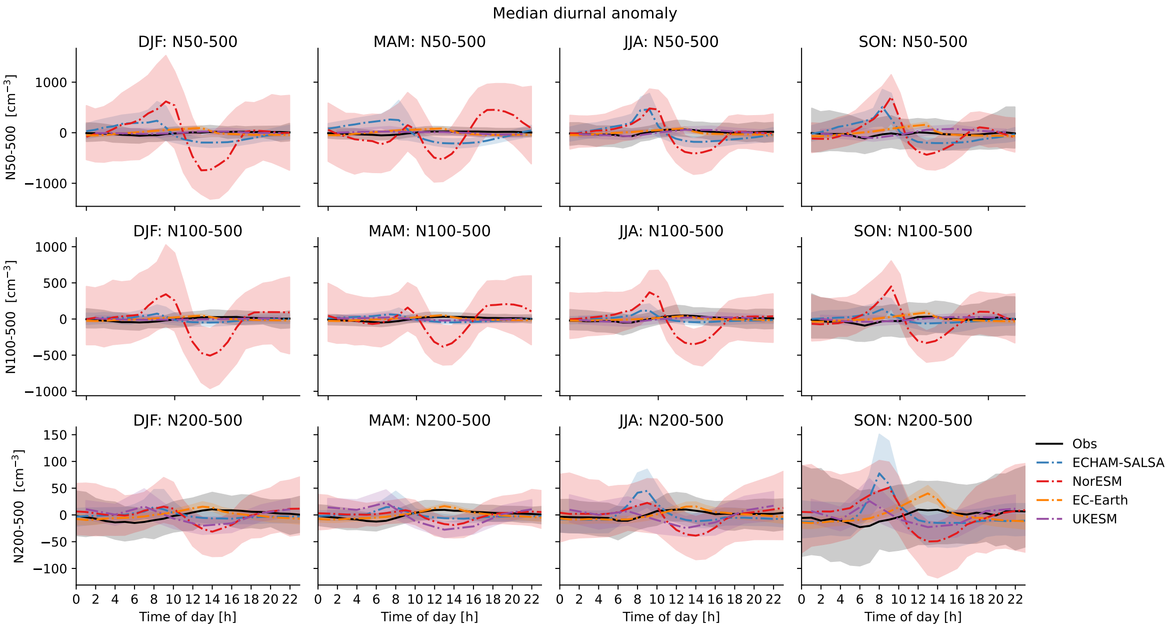

Figure S54. ATTO: Diurnal variation in N_{50} (first row), N_{100} (second row) and N_{200} (third row) in DJF (first column), MAM (second column), JJA (third column) and SON (last column). The data is in hourly resolution for observations and all models except UKESM for which we had 3 hourly output.

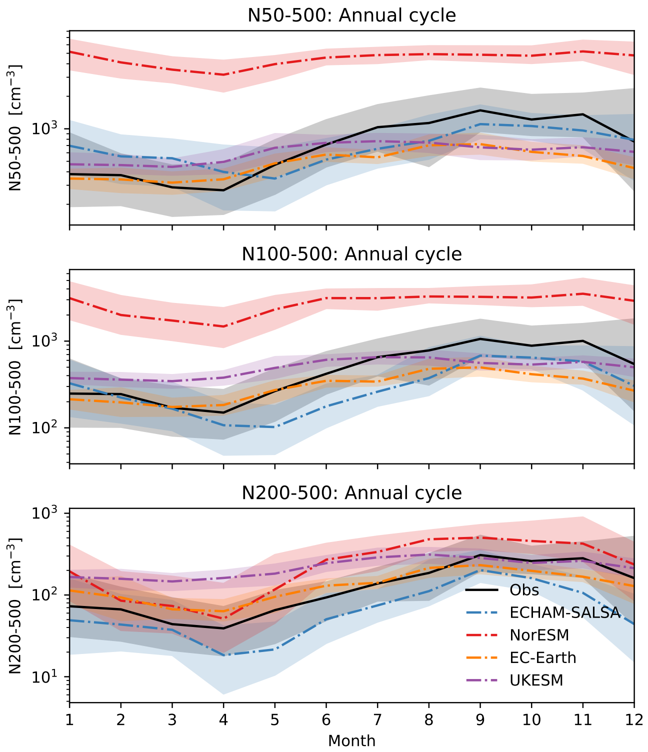

Figure S55. ATTO: Monthly median N_{50} (top), N_{100} (middle) and N_{200} (bottom). Lines signify median as calculated from all available data and shading signifies the 16th to 84th percentiles from data in hourly resolution, except UKESM for which we use 3 hourly resolution. Model values included only if the corresponding time exists in the observations. This computation method entails some more weight to years with more data available.

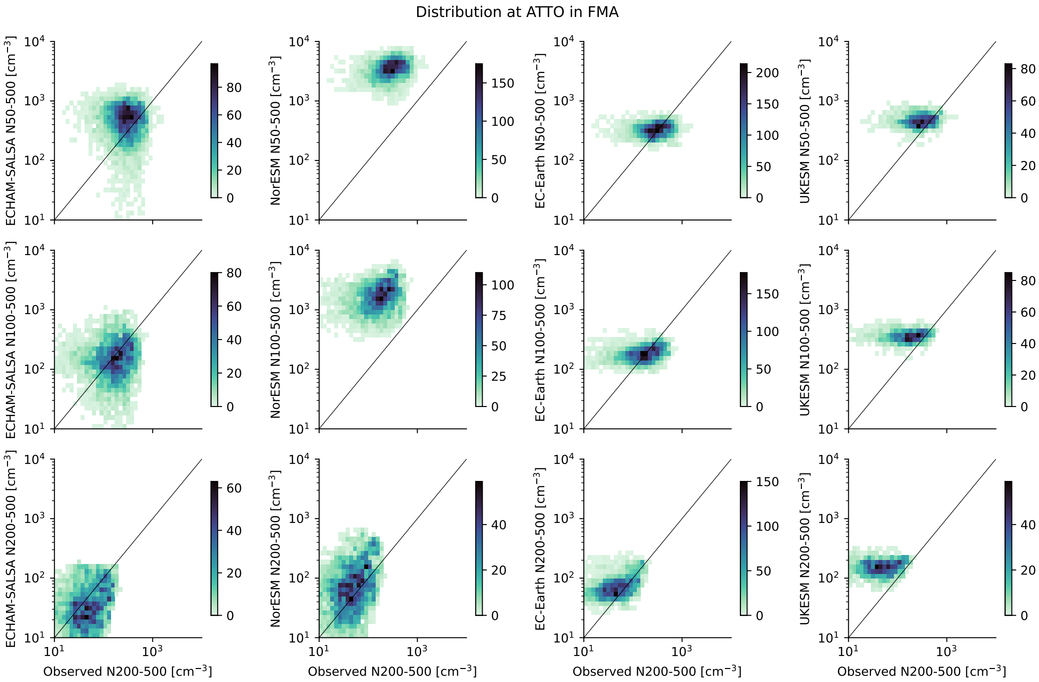

Figure S56. ATTO: Distribution of modelled versus observed N_{50} (first row), N_{100} (second row) and N_{200} (third row) in February–April. The data is in hourly resolution for observations and all models except UKESM for which we had 3 hourly output.

S10.3 ATTO: Time series N50, N100, N200

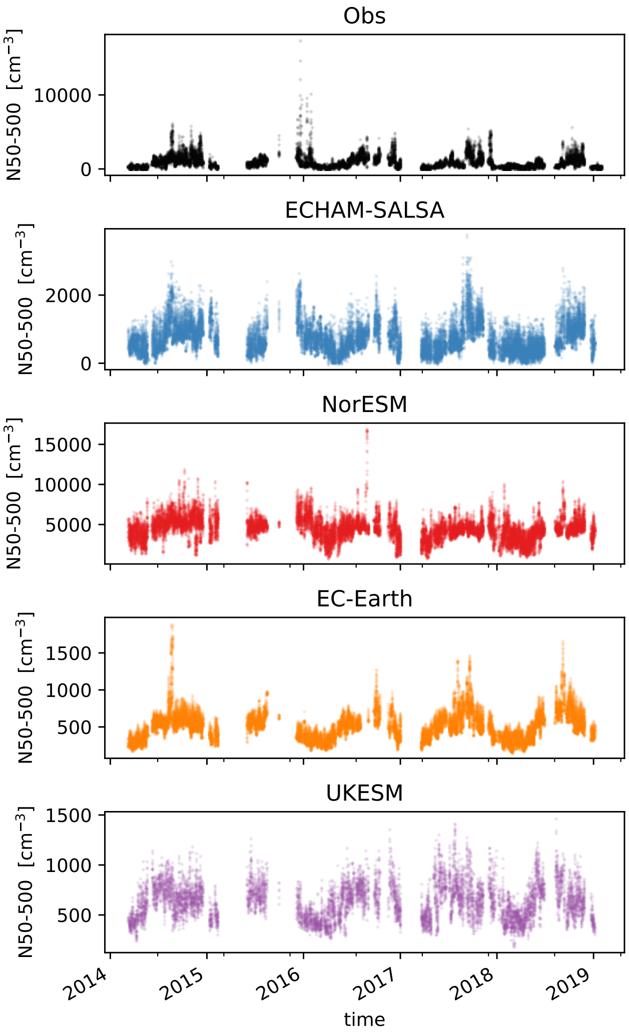

Figure S57. ATTO: Time series of models and observations of N_{50}. The modelled data is shown only when there is not missing data in the observations. The data is in hourly resolution for observations and all models except UKESM for which we had 3 hourly output.

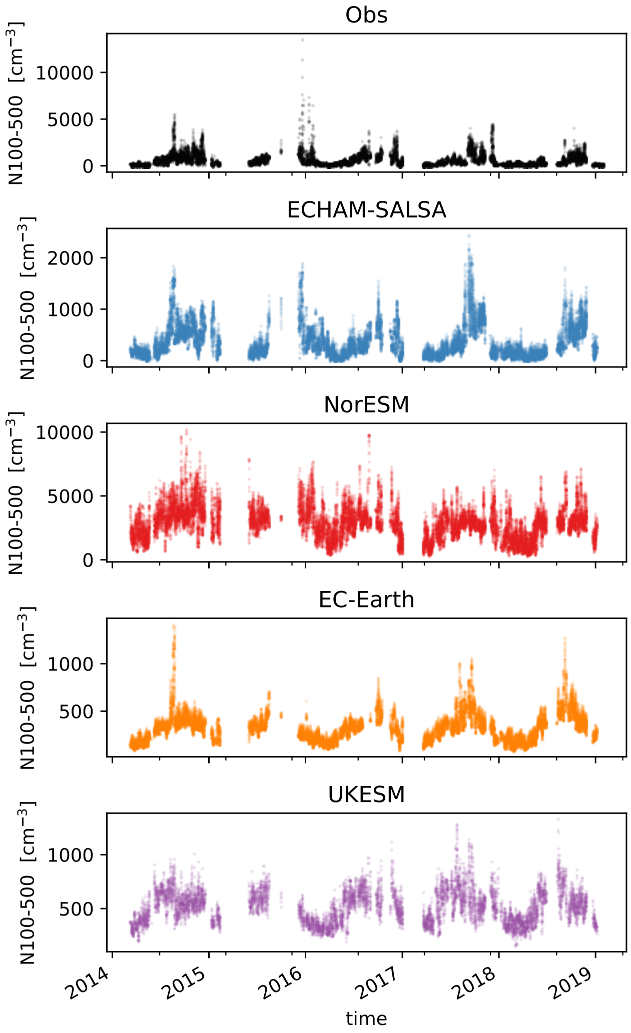

Figure S58. ATTO: Time series of models and observations of N_{100}. The modelled data is shown only when there is not missing data in the observations. The data is in hourly resolution for observations and all models except UKESM for which we had 3 hourly output.

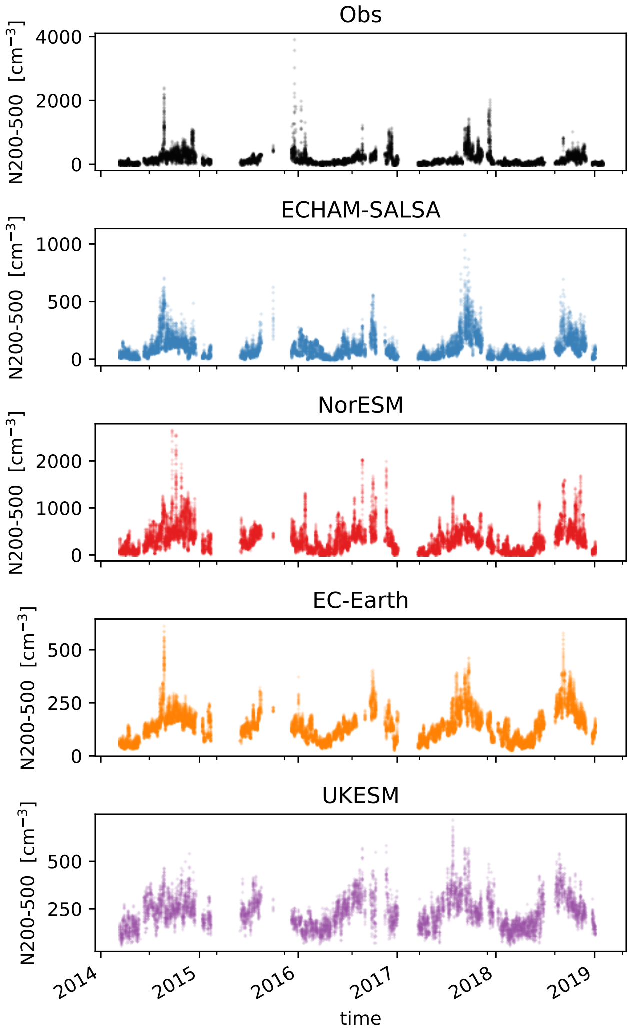

Figure S59. ATTO: Time series of models and observations of N_{200}. The modelled data is shown only when there is not missing data in the observations. The data is in hourly resolution for observations and all models except UKESM for which we had 3 hourly output.

S11 Model descriptions

S11.1 The Norwegian Earth System model

The Norwegian Earth System model version 2 [NorESM2 8, 9, 10] is based on the Community Earth System Model [CESM2 11], but has a different ocean model (Bergen Layered Ocean Model, BLOM) and significant changes to the atmospheric component, including a different chemistry and aerosol model ([12]). In the simulations in this paper, we use sea surface temperatures and sea ice data based on the Hadley Centre Sea Ice and Sea Surface Temperature data set [HADISST, 13] as described in [14], so the ocean component is not in use. The atmospheric component used in NorESM, CAM6-Nor, is based on the Community Atmospheric model version 6 [CAM6, see e.g 15]) but has a very different aerosol scheme called OsloAero6 [described below, see also 16] and also include improvements to the local dry and moist energy conservation, angular momentum conservation and in the computation of air-sea fluxes and deep convection. As in CAM6, the cloud macrophysics scheme in CAM6-Nor, is the Cloud Layers Unified by Binomials model [CLUBB; 15]. For the microphysics in shallow convection and stratiform clouds, the two-moment bulk scheme MG2, [17], is used. Finally, the representation of microphysics in deep convective clouds is based on [18]. Cloud droplet activation is calculated using the Abdul-Razzak scheme [19]. More details on the treatment of clouds in CAM6 and CAM6-Nor can be found in [15].

OsloAero6 is a production-tagged aerosol and chemistry model which keeps track of the source and origin of the aerosols through their lifetime. The most notable difference to other aerosol schemes is how the aerosol tracers are divided into

NorESM	ECHAM-SALSA	EC-Earth	UKESM
OH + IP → 5%LVOC<br>NO_3 + IP → 5%LVOC<br>O_3 + IP → 5%LVOC<br>OH+MT → 15%LVOC<br>NO_3 + MT → 15%LVOC<br>O_3 + MT → 15%ELVOC<br>Both LVOC and ELVOC<br>are treated as essentially<br>non-volatile, but ELVOC<br>can participate in<br>NPF and early growth.	3 VBS classes with saturation<br>vapor concentrations<br>at STP of 0, 1, and 10 μg/m3<br>denoted V_0, V_1, V_{10}, respectively<br>(OH/O_3/NO_3) + MT →10%V_0<br>+ 3.7%V_1 + 8.5%V_{10}<br>(OH/O_3/NO_3) + IP →2.95%V_1<br>+ 4.53%V_{10}.<br>OH + benzene → 37%V_0<br>OH + toluene → 36%V_0<br>OH + xylene →30%V_0	OH+IP → 0.97%LVOC<br>+ 0.03%ELVOC<br>O_3 + IP → 0.99%LVOC<br>+ 0.01%ELVOC<br>OH+MT → 14%LVOC<br>+ 1%ELVOC<br>O_3+MT → 10%LVOC<br>+ 5%ELVOC<br>Both LVOC and ELVOC<br>are treated as essentially<br>non-volatile, but<br>ELVOC can participate in<br>NPF and early growth.	OH+MT → 26%LVOC<br>O_3 + MT → 26%LVOC<br>NO_3 + MT → 26%LVOC<br>Treated as essentially<br>non-volatile<br>during condensation.

Table S4. Overview of molar SOA yields in the models

NorESM	ECHAM-SALSA	EC-Earth	UKESM
OH + IP → 12.33%LVOC<br>NO_3 + IP → 12.33%LVOC<br>O_3 + IP → 12.33%LVOC<br>OH+MT → 18.5%LVOC<br>NO_3 + MT → 18.5%LVOC<br>O_3 + MT → 18.5%ELVOC<br>Both LVOC and ELVOC<br>are treated as essentially<br>non-volatile, but ELVOC<br>can participate in<br>NPF and early growth.	3 VBS classes with saturation<br>vapor concentrations<br>at STP of 0, 1, and 10 μg/m3<br>denoted V_0, V_1, V_{10}, respectively<br>(OH/O_3/NO_3) + MT →10%V_0<br>+ 3.7%V_1 + 8.5%V_{10}<br>(OH/O_3/NO_3) + IP →5.9%V_1<br>+ 9.06%V_{10}.<br>OH + benzene → 76.2%V_0<br>OH + toluene → 53.2%V_0<br>OH + xylene →38.5%V_0	OH+IP → 3.3%LVOC<br>+ 0.11%ELVOC<br>O_3 + IP → 3.37%LVOC<br>+ 0.036%ELVOC<br>OH+MT → 23.8%LVOC<br>+ 1.8%ELVOC<br>O_3+MT → 17%LVOC<br>+ 9.1%ELVOC<br>Both LVOC and ELVOC<br>are treated as essentially<br>non-volatile, but<br>ELVOC can participate in<br>NPF and early growth.	OH+MT → 28.7%LVOC<br>O_3 + MT → 28.7%LVOC<br>NO_3 + MT → 28.7%LVOC<br>Treated as essentially<br>non-volatile<br>during condensation.

Table S5. Overview of SOA mass yields in the models

"background"- and "process" tracers. The background tracers determine the number of particles and form an initial size distribution of log-normal modes. The process tracers then alter this initial distribution and their chemical composition based on look-up-tables from the offline scheme AeroTab [see e.g. 16] – thus meaning that the resulting size distribution need not be log-normal. Examples of the background tracers are dust, sea salt, primary organics, black carbon, coated black carbon, primary sulphate and so on. Examples of process tracers are secondary organic condensate, secondary sulphate condensate and correspondingly coagulate of each of these. The mass of each of the tracers is tracked, and the optical properties and the size distributions for cloud activation are calculated using a look-up table [see 16].

The land model used is the Community Land Model version 5 [CLM5, 20], in BGC (bio-geo-chemisty) model and with prognostic crop. This means that the vegetation is allowed to respond to meteorological conditions, soil moisture, nutrient availability and so on by growing more or less dense (leaf area index can change), but that the distribution (the land area covered by each vegetation type) of the vegetation remains set (not dynamic vegetation). The emissions of BVOCs in CLM5 are calculated using the Model of Emissions of Gases and Aerosols from Nature version 2.1[MEGAN2.1 4], which is incorporated into CLM5. The simulations for this study were run with nudged meteorology (horizontal wind and surface pressure) to ERA-Interim [21] using a relaxation time of 6 h [22].

S11.2 EC-Earth

The simulations were run with EC-Earth3-AERCHEM configuration of EC-Earth3.3.4. The model uses the Integrated Forecasting System (IFS) cycle 36r4 as the General circulation model (GCM), together with the surface-exchange land model H-TESSEL [23] and the chemistry model Tracer Model 5- Massively Parallel (TM5-MP) version 1.1, with modified chemistry from CB05 [24, 25, 26]. For this study, CB05 was modified so as to read daily $0.5^\circ \times 0.5^\circ$ emissions of monoterpene and isoprene generated by the 2nd generation dynamical global vegetation model LPJ-GUESS (v4.1) [27], replacing the default MEGAN based monthly emission files in TM5 for the two BVOC precursors. The IFS model has a T255 (0.7°) spectral truncation with N128 reduced Gaussian grid, on 91 vertical levels. TM5 has a $3^\circ \times 2^\circ$ grid on 34 vertical levels with the same hybrid sigma-pressure levels as IFS but with lower resolution. The coupling between IFS and TM5 is processed though OASIS3 [28]. Even though LPJ-GUESS 4.0 [27] is the dynamical vegetation model embedded in EC-Earth3, there isn't currently a set-up for the coupling to the CTM TM5 within EC-Earth3.3.4. LPJ-GUESS 4.1 was instead run offline with daily ERA-Interim forcing to provide daily emissions. In EC-Earth3, the IFS output variables are instantaneous values with model time-step of 45 minutes, and output is available only as 3 hourly. For TM5 we get instantaneous values with model timestep of 1 hour with 1-hourly output. The simulations were nudged using ERA-Interim data for divergence, vorticity, and surface pressure, and the nudging was applied with a 6-hour relaxation time.

S11.3 ECHAM-SALSA

The latest stable version ECHAM-HAMMOZ (ECHAM6.3-HAM2.3-MOZ1.0) is a three-dimensional aerosol-chemistry-climate model [29, 30]. ECHAM-HAMMOZ consists of general circulation model (GCM) ECHAM version 6.3 (ECHAM6) [30], Hamburg Aerosol Module version 2.3 (HAM) [31, 32] and Model for Ozone and Related chemical Tracers version 1.0 (MOZ) [33]. The GCM ECHAM6 solves equations for divergence, vorticity and surface pressure [34]. These variables were nudged towards ERA-Interim data from European Centre for Medium-Range Weather Forecasts (ECMWF) [35, 36]. HAM is used to calculate all the aerosol processes within ECHAM-HAMMOZ. The processes included in the calculations are microphysics, radiation, emissions and deposition [32]. The aerosol size distribution in HAM can be represented using comprehensive parameterization using both modal and sectional methods [31, 32]. In this study the Sectional Aerosol module for Large Scale Application (SALSA) was used to represent the aerosol size distribution in the ECHAM-HAMMOZ simulations (hereafter referred to as ECHAM-SALSA) [31]. The vegetation model used in ECHAM-SALSA is JSBACH version 3 and they are coupled through surface exchange of heat, momentum and mass [37, 38]. JSBACH uses three non-dynamic vegetation pools for living vegetation and it simulates processes such as natural and anthropogenic disturbances, shedding of leaves, and grazing leading to losses from the vegetation pools [39]. The BVOC emissions in ECHAM-SALSA are calculated using MEGAN2.1 and the treatment of organic compounds involved in SOA formation is based on Volatility Basis Set (VBS) approach [4, 40]. The gas-particle partitioning of VBS species is calculated assuming non-equilibrium partitioning and solving condensation equations according to [41].

S11.4 UKESM

The United Kingdom Earth System Model (UKESM1, [42, 43]) is a global climate model based upon the HadGEM3-GC3.1 core physical dynamical model of the atmosphere, land, ocean and sea ice systems [44, 45, 46] which constitute the UK's contribution to the Coupled Model Intercomparison Project Phase 6 (CMIP 6) [47]. UKESM1 simulations are built using the atmosphere-only configuration of the Atmospheric Model Intercomparison Project (AMIP) that work with a time-evolving sea surface temperature, sea ice and prescribed marine biogenic emissions from a fully coupled model simulation. The N96L85 model configuration is used in this study, which is $1.875^\circ \times 1.25^\circ$ longitude–latitude, corresponding to a near-equator

horizontal resolution of approximately 208 km. The vertical model levels are divided into 50 levels between Earth's surface and 18 km and 35 levels between 18 and 85 km. The model output fields are extracted at high temporal resolution (3-hourly output) to reduce model sampling errors when evaluating against observations [48]. A nudged configuration was applied, where horizontal winds (but not temperature) in the model are relaxed towards fields from ERA-Interim reanalysis [49, 50].The nudging is applied between model levels 12 and 80 with a relaxation time constant of 6 hours (which is equal to the ERA-Interim reanalysis temporal resolution fields). Anthropogenic and biomass-burning emissions are taken from [51] and [52] while greenhouse gases concentrations derive from [51]. Atmospheric composition is simulated using the chemistry-aerosol component model of UKESM, the UK Chemistry and Aerosol model (UKCA) [53, 54, 55]. Within UKCA, the microphysical processes of new particle formation (NPF), condensation, coagulation, wet scavenging, dry deposition and cloud processing are determined by the Global Model of Aerosol Processes, GLOMAP model [43, 56]. The UKCA stratospheric–tropospheric (StratTrop) chemistry scheme is fully integrated with aerosol chemistry in UKESM1 [54, 55, 57] The chemical oxidants hydroxyl radical (OH), ozone (O_3) and nitrate radical (NO_3) are gas-phase aerosol precursors which are interactively simulated with the respective production and loss mechanisms. Isoprene and monoterpenes emission mechanisms are simulated using iBVOC, an interactive biogenic VOC (BVOC) terrestrial emission scheme [58, 59]. For isoprene the emission scheme in iBVOC is from [58] and for monoterpene they are from [60]. Isoprene is not included in the formation of SOA due to the more complex formation mechanism. The emissions of biogenic isoprene are based on a simplified mechanistic scheme of [58]. The monoterpenes emission parameterisation follows [60]. Land-based monoterpenes are emitted via gas-phase and produce secondary organic aerosol (SOA) through gas-phase oxidation processes driven by OH, NO_3 and O_3. The molar yield of SOA from these reactions is 26 %, which amounts to mass yield of 28.7 % [43]. The UKESM1 terrestrial biogeochemistry and land surface are processed by the Joint UK Land Environment Simulator (JULES) model [61, 62].

S12 Model data post-processing

We use only CWP values below 800 gm^{-2} at ATTO because the distribution for UKESM has an unrealistically long tail (see Fig. S33b).

S12.0.1 EC-Earth

- Corrected particle number concentrations and OA mass concentrations to standard temperature and pressure (STP) for EC-Earth was done assuming standard pressure, since we do not have ambient pressure as a model output. The impact is maximally 2–3 percent. Additionally, temperature is from the IFS component and is only updates each 3 hour. In order to use temperature in the conversion to STP we therefore interpolate with quadratic interpolation the temperature for specifically this calculation.
- We mask cloud data where the cloud top cloud fraction is less than 10 % to avoid skewing towards times with very little cloud.
- To extract only the liquid clouds, we mask data points where ice water path is more than 5 % of the total cloud water path.
- EC-Earth does not have cloud top values as default output, so these are extracted by taking the highest gridbox in each column where the cloud time is 1 and cloud fraction is above zero. We mask values where the cloud top fraction is below 10% and select for liquid cloud by including only grid cells where the liquid cloud fraction of the cloud water path is more than 80%.

S12.0.2 UKESM

- Corrected particle number concentrations and OA mass concentrations to STP for UKESM was done assuming standard pressure, since we do not have ambient pressure as a model output. The impact will maximally be a 2–3 percent.
- For UKESM, we use the second to bottom level at ATTO because it is more consistent with the height of the measurements. This is in contrast to the other models which have lower vertical resolution at the surface.
- To extract only the liquid clouds, we mask data points where ice water path is more than 5 % of the total cloud water path.
- We mask data points where cloud top cloud fraction is less than 10 % to avoid skewing towards times with very little cloud.

S12.0.3 NorESM

- We correct particle number concentrations to be STP, while OA mass concentrations are calculated from mass mixing ratios using STP.

- To extract only the liquid clouds, we mask data points where ice water path is more than 5 % of the total cloud water path.
- We mask data points where cloud top cloud fraction is less than 10 % to avoid skewing towards times with very little cloud.
- To compute the COT, we sum over total grid box cloud optical thickness in the column (TOT_CLD_VISTAU), and divide by the total cloud (CLDTOT) to get in-cloud COT.
- Similarly, the model output total gridbox LWP is divided by CLDTOT to get in-cloud values.

S12.0.4 ECHAM-SALSA

- Particle number concentrations and OA mass concentrations are calculated from mass mixing ratios using STP.
- To extract only the liquid clouds, we remove cases where the cloud top temperature is below $-15\,^{\circ}$C.
- We only use values where there is a cloud more than 10% of the time in the column.
- We mask cloud data where the cloud top cloud fraction is less than 10 % to avoid skewing towards times with very little cloud (this is performed on the hourly resolution).
- In ECHAM-SALSA COT and CWP are both output as instantaneous values and we divide by the column maximum of the cloud time over the column (the fraction of time there has been a cloud in the column).

S13 Supplementary references

References

1. Yli-Juuti, T. *et al.* Significance of the organic aerosol driven climate feedback in the boreal area. *Nat. Commun.* **12**, 5637, DOI: 10.1038/s41467-021-25850-7 (2021).

2. Artaxo, P. *et al.* Tropical and Boreal Forest – Atmosphere Interactions: A Review. *Tellus B: Chem. Phys. Meteorol.* **74**, 24–163, DOI: 10.16993/tellusb.34 (2022).

3. Forster, P. *et al.* The Earth's energy budget, climate feedbacks, and climate sensitivity. In Masson-Delmotte, V. *et al.* (eds.) *Climate Change 2021: The Physical Science Basis. Contribution of Working Group I to the Sixth Assessment Report of the Intergovernmental Panel on Climate Change* (Cambridge University Press, 2021).

4. Guenther, A. B. *et al.* The Model of Emissions of Gases and Aerosols from Nature version 2.1 (MEGAN2.1): An extended and updated framework for modeling biogenic emissions. *Geosci. Model. Dev.* **5**, 1471–1492, DOI: 10.5194/gmd-5-1471-2012 (2012).

5. Peñuelas, J. & Staudt, M. BVOCs and global change. *Trends Plant Sci.* **15**, 133–144, DOI: 10.1016/j.tplants.2009.12.005 (2010).

6. McFiggans, G. *et al.* Secondary organic aerosol reduced by mixture of atmospheric vapours. *Nature* **565**, 587, DOI: 10.1038/s41586-018-0871-y (2019).

7. Sporre, M. K. *et al.* Large difference in aerosol radiative effects from BVOC-SOA treatment in three Earth system models. *Atmospheric Chem. Phys.* **20**, 8953–8973, DOI: 10.5194/acp-20-8953-2020 (2020).

8. Bentsen, M. *et al.* The Norwegian Earth System Model, NorESM1-M – Part 1: Description and basic evaluation of the physical climate. *Geosci. Model. Dev.* **6**, 687–720, DOI: 10.5194/gmd-6-687-2013 (2013).

9. Kirkevåg, A. *et al.* Aerosol–climate interactions in the Norwegian Earth System Model – NorESM1-M. *Geosci. Model. Dev.* **6**, 207–244, DOI: 10.5194/gmd-6-207-2013 (2013).

10. Iversen, T. *et al.* The Norwegian Earth System Model, NorESM1-M – Part 2: Climate response and scenario projections. *Geosci. Model. Dev.* **6**, 389–415, DOI: 10.5194/gmd-6-389-2013 (2013).

11. Danabasoglu, G. *et al.* The Community Earth System Model Version 2 (CESM2). *J. Adv. Model. Earth Syst.* **12**, e2019MS001916, DOI: 10.1029/2019MS001916 (2020).

12. Seland, Ø. *et al.* Overview of the Norwegian Earth System Model (NorESM2) and key climate response of CMIP6 DECK, historical, and scenario simulations. *Geosci. Model. Dev.* **13**, 6165–6200, DOI: 10.5194/gmd-13-6165-2020 (2020).

13. Rayner, N. A. *et al.* Global analyses of sea surface temperature, sea ice, and night marine air temperature since the late nineteenth century. *J. Geophys. Res. Atmospheres* **108**, DOI: 10.1029/2002JD002670 (2003).

14. Hurrell, J. W., Hack, J. J., Shea, D., Caron, J. M. & Rosinski, J. A New Sea Surface Temperature and Sea Ice Boundary Dataset for the Community Atmosphere Model. *J. Clim.* **21**, 5145–5153, DOI: 10.1175/2008JCLI2292.1 (2008).

15. Bogenschutz, P. A. *et al.* The path to CAM6: Coupled simulations with CAM5.4 and CAM5.5. *Geosci. Model. Dev.* **11**, 235–255, DOI: 10.5194/gmd-11-235-2018 (2018).

16. Kirkevåg, A. *et al.* A production-tagged aerosol module for Earth system models, OsloAero5.3 – extensions and updates for CAM5.3-Oslo. *Geosci. Model. Dev.* **11**, 3945–3982, DOI: 10.5194/gmd-11-3945-2018 (2018).

17. Gettelman, A. & Morrison, H. Advanced Two-Moment Bulk Microphysics for Global Models. Part I: Off-Line Tests and Comparison with Other Schemes. *J. Clim.* **28**, 1268–1287, DOI: 10.1175/JCLI-D-14-00102.1 (2015).

18. Zhang, G. J. & McFarlane, N. A. Sensitivity of climate simulations to the parameterization of cumulus convection in the Canadian climate centre general circulation model. *Atmosphere-Ocean* **33**, 407–446, DOI: 10.1080/07055900.1995.9649539 (1995).

19. Abdul-Razzak, H. & Ghan, S. J. A parameterization of aerosol activation: 2. Multiple aerosol types. *J. Geophys. Res. Atmospheres* **105**, 6837–6844, DOI: 10.1029/1999JD901161 (2000).

20. Lawrence, D. M. *et al.* The Community Land Model Version 5: Description of New Features, Benchmarking, and Impact of Forcing Uncertainty. *J. Adv. Model. Earth Syst.* **11**, 4245–4287, DOI: 10.1029/2018MS001583 (2019).

21. European Centre for Medium-range Weather Forecast (ECMWF) (2011).

22. Kooperman, G. J. *et al.* Constraining the influence of natural variability to improve estimates of global aerosol indirect effects in a nudged version of the Community Atmosphere Model 5. *J. Geophys. Res. Atmospheres* **117**, DOI: 10.1029/2012JD018588 (2012).

23. Balsamo, G. *et al.* A Revised Hydrology for the ECMWF Model: Verification from Field Site to Terrestrial Water Storage and Impact in the Integrated Forecast System. *J. Hydrometeorol.* **10**, 623–643, DOI: 10.1175/2008JHM1068.1 (2009).

24. Krol, M. *et al.* The two-way nested global chemistry-transport zoom model TM5: Algorithm and applications. *Atmospheric Chem. Phys.* **5**, 417–432, DOI: 10.5194/acp-5-417-2005 (2005).

25. van Noije, T. P. C. *et al.* Simulation of tropospheric chemistry and aerosols with the climate model EC-Earth. *Geosci. Model. Dev.* **7**, 2435–2475, DOI: 10.5194/gmd-7-2435-2014 (2014).

26. Williams, J. E., Boersma, K. F., Le Sager, P. & Verstraeten, W. W. The high-resolution version of TM5-MP for optimized satellite retrievals: Description and validation. *Geosci. Model. Dev.* **10**, 721–750, DOI: 10.5194/gmd-10-721-2017 (2017).

27. Smith, B. *et al.* Implications of incorporating N cycling and N limitations on primary production in an individual-based dynamic vegetation model. *Biogeosciences* **11**, 2027–2054, DOI: 10.5194/bg-11-2027-2014 (2014).

28. Valcke, S. The OASIS3 coupler: A European climate modelling community software. *Geosci. Model. Dev.* **6**, 373–388, DOI: 10.5194/gmd-6-373-2013 (2013).

29. Schultz, M. G. *et al.* The chemistry–climate model ECHAM6.3-HAM2.3-MOZ1.0. *Geosci. Model. Dev.* **11**, 1695–1723, DOI: 10.5194/gmd-11-1695-2018 (2018).

30. Stevens, B. *et al.* Atmospheric component of the MPI-M Earth System Model: ECHAM6. *J. Adv. Model. Earth Syst.* **5**, 146–172, DOI: 10.1002/jame.20015 (2013).

31. Kokkola, H. *et al.* SALSA2.0: The sectional aerosol module of the aerosol–chemistry–climate model ECHAM6.3.0-HAM2.3-MOZ1.0. *Geosci. Model. Dev.* **11**, 3833–3863, DOI: 10.5194/gmd-11-3833-2018 (2018).

32. Tegen, I. *et al.* The global aerosol–climate model ECHAM6.3–HAM2.3 – Part 1: Aerosol evaluation. *Geosci. Model. Dev.* **12**, 1643–1677, DOI: 10.5194/gmd-12-1643-2019 (2019).

33. Schultz, M. G. *et al.* The chemistry–climate model ECHAM6.3-HAM2.3-MOZ1.0. *Geosci. Model. Dev.* **11**, 1695–1723, DOI: 10.5194/gmd-11-1695-2018 (2018).

34. Stier, P. *et al.* The aerosol-climate model ECHAM5-HAM. *Atmospheric Chem. Phys.* **5**, 1125–1156, DOI: 10.5194/acp-5-1125-2005 (2005).

35. Berrisford, P. *et al.* The ERA-Interim archive Version 2.0 (2011).

36. Simmons, A. J., Burridge, D. M., Jarraud, M., Girard, C. & Wergen, W. The ECMWF medium-range prediction models development of the numerical formulations and the impact of increased resolution. *Meteorol. Atmospheric Phys.* **40**, 28–60, DOI: 10.1007/BF01027467 (1989).

37. Giorgetta, M. A. *et al.* Climate and carbon cycle changes from 1850 to 2100 in MPI-ESM simulations for the Coupled Model Intercomparison Project phase 5. *J. Adv. Model. Earth Syst.* **5**, 572–597, DOI: 10.1002/jame.20038 (2013).

38. Mauritsen, T. *et al.* Developments in the MPI-M Earth System Model version 1.2 (MPI-ESM1.2) and Its Response to Increasing CO2. *J. Adv. Model. Earth Syst.* **11**, 998–1038, DOI: 10.1029/2018MS001400 (2019).

39. Thum, T. *et al.* Evaluating two soil carbon models within the global land surface model JSBACH using surface and spaceborne observations of atmospheric CO_2. *Biogeosciences* **17**, 5721–5743, DOI: 10.5194/bg-17-5721-2020 (2020).

40. Koo, B., Knipping, E. & Yarwood, G. 1.5-Dimensional volatility basis set approach for modeling organic aerosol in CAMx and CMAQ. *Atmospheric Environ.* **95**, 158–164, DOI: 10.1016/j.atmosenv.2014.06.031 (2014).

41. Jacobson, M. Z. *Fundamentals of Atmospheric Modeling: Second Edition* (Cambridge University Press, Cambridge, 2005), 2 edn.

42. Sellar, A. A. *et al.* UKESM1: Description and Evaluation of the U.K. Earth System Model. *J. Adv. Model. Earth Syst.* **11**, 4513–4558, DOI: 10.1029/2019MS001739 (2019).

43. Mulcahy, J. P. *et al.* Description and evaluation of aerosol in UKESM1 and HadGEM3-GC3.1 CMIP6 historical simulations. *Geosci. Model. Dev.* **13**, 6383–6423, DOI: 10.5194/gmd-13-6383-2020 (2020).

44. Ridley, J. K. *et al.* The sea ice model component of HadGEM3-GC3.1. *Geosci. Model. Dev.* **11**, 713–723, DOI: 10.5194/gmd-11-713-2018 (2018).

45. Storkey, D. *et al.* UK Global Ocean GO6 and GO7: A traceable hierarchy of model resolutions. *Geosci. Model. Dev.* **11**, 3187–3213, DOI: 10.5194/gmd-11-3187-2018 (2018).

46. Walters, D. *et al.* The Met Office Unified Model Global Atmosphere 6.0/6.1 and JULES Global Land 6.0/6.1 configurations. *Geosci. Model. Dev.* **10**, 1487–1520, DOI: 10.5194/gmd-10-1487-2017 (2017).

47. Eyring, V. *et al.* Overview of the Coupled Model Intercomparison Project Phase 6 (CMIP6) experimental design and organization. *Geosci. Model. Dev.* **9**, 1937–1958, DOI: 10.5194/gmd-9-1937-2016 (2016).

48. Schutgens, N. A. J. *et al.* Will a perfect model agree with perfect observations? The impact of spatial sampling. *Atmospheric Chem. Phys.* **16**, 6335–6353, DOI: 10.5194/acp-16-6335-2016 (2016).

49. Dee, D. P. *et al.* The ERA-Interim reanalysis: Configuration and performance of the data assimilation system. *Q. J. Royal Meteorol. Soc.* **137**, 553–597, DOI: 10.1002/qj.828 (2011).

50. Telford, P. J., Braesicke, P., Morgenstern, O. & Pyle, J. A. Technical Note: Description and assessment of a nudged version of the new dynamics Unified Model. *Atmospheric Chem. Phys.* **8**, 1701–1712, DOI: 10.5194/acp-8-1701-2008 (2008).

51. Hoesly, R. M. *et al.* Historical (1750–2014) anthropogenic emissions of reactive gases and aerosols from the Community Emissions Data System (CEDS). *Geosci. Model. Dev.* **11**, 369–408, DOI: 10.5194/gmd-11-369-2018 (2018).

52. van Marle, M. J. E. *et al.* Historic global biomass burning emissions for CMIP6 (BB4CMIP) based on merging satellite observations with proxies and fire models (1750–2015). *Geosci. Model. Dev.* **10**, 3329–3357, DOI: 10.5194/gmd-10-3329-2017 (2017).

53. Morgenstern, O. *et al.* Review of the global models used within phase 1 of the Chemistry–Climate Model Initiative (CCMI). *Geosci. Model. Dev.* **10**, 639–671, DOI: 10.5194/gmd-10-639-2017 (2017).

54. O'Connor, F. M. *et al.* Evaluation of the new UKCA climate-composition model – Part 2: The Troposphere. *Geosci. Model. Dev.* **7**, 41–91, DOI: 10.5194/gmd-7-41-2014 (2014).

55. Archibald, A. T. *et al.* Description and evaluation of the UKCA stratosphere–troposphere chemistry scheme (StratTrop vn 1.0) implemented in UKESM1. *Geosci. Model. Dev.* **13**, 1223–1266, DOI: 10.5194/gmd-13-1223-2020 (2020).

56. Mann, G. W. *et al.* Description and evaluation of GLOMAP-mode: A modal global aerosol microphysics model for the UKCA composition-climate model. *Geosci. Model. Dev.* **3**, 519–551, DOI: 10.5194/gmd-3-519-2010 (2010).

57. Morgenstern, O. *et al.* Evaluation of the new UKCA climate-composition model – Part 1: The stratosphere. *Geosci. Model. Dev.* **2**, 43–57, DOI: 10.5194/gmd-2-43-2009 (2009).

58. Pacifico, F. *et al.* Evaluation of a photosynthesis-based biogenic isoprene emission scheme in JULES and simulation of isoprene emissions under present-day climate conditions. *Atmospheric Chem. Phys.* **11**, 4371–4389, DOI: 10.5194/acp-11-4371-2011 (2011).

59. Pacifico, F., Folberth, G. A., Jones, C. D., Harrison, S. P. & Collins, W. J. Sensitivity of biogenic isoprene emissions to past, present, and future environmental conditions and implications for atmospheric chemistry. *J. Geophys. Res. Atmospheres* **117**, DOI: 10.1029/2012JD018276 (2012).

60. Guenther, A. *et al.* A global model of natural volatile organic compound emissions. *J. Geophys. Res. Atmospheres* **100**, 8873–8892, DOI: 10.1029/94JD02950 (1995).

61. Best, M. J. *et al.* The Joint UK Land Environment Simulator (JULES), model description – Part 1: Energy and water fluxes. *Geosci. Model. Dev.* **4**, 677–699, DOI: 10.5194/gmd-4-677-2011 (2011).

62. Clark, D. B. *et al.* The Joint UK Land Environment Simulator (JULES), model description – Part 2: Carbon fluxes and vegetation dynamics. *Geosci. Model. Dev.* **4**, 701–722, DOI: 10.5194/gmd-4-701-2011 (2011).
